# Delivery of sexual and reproductive health interventions in conflict settings: a systematic review

**DOI:** 10.1136/bmjgh-2019-002206

**Published:** 2020-07-21

**Authors:** Mariella Munyuzangabo, Dina Sami Khalifa, Michelle F Gaffey, Mahdis Kamali, Fahad J Siddiqui, Sarah Meteke, Shailja Shah, Reena P Jain, Daina Als, Amruta Radhakrishnan, Anushka Ataullahjan, Zulfiqar A Bhutta

**Affiliations:** 1 Centre for Global Child Health, The Hospital for Sick Children, Toronto, Ontario, Canada; 2 Health Services and Systems Research, Duke-NUS Graduate Medical School, Singapore; 3 Center of Excellence in Women and Child Health, Aga Khan University, Karachi, Pakistan

**Keywords:** health systems evaluation, child health, mental health & psychiatry, systematic review, public health

## Abstract

**Background:**

It is essential to provide comprehensive sexual and reproductive health (SRH) interventions to women affected by armed conflict, but there is a lack of evidence on effective approaches to delivering such interventions in conflict settings. This review synthesised the available literature on SRH intervention delivery in conflict settings to inform potential priorities for further research and additional guidance development.

**Methods:**

We searched MEDLINE, Embase, CINAHL and PsycINFO databases using terms related to conflict, women and children, and SRH. We searched websites of 10 humanitarian organisations for relevant grey literature. Publications reporting on conflict-affected populations in low-income and middle-income countries and describing an SRH intervention delivered during or within 5 years after the end of a conflict were included. Information on population, intervention and delivery characteristics were extracted and narratively synthesised. Quantitative data on intervention coverage and effectiveness were tabulated, but no meta-analysis was undertaken.

**Results:**

110 publications met our eligibility criteria. Most focused on sub-Saharan Africa and displaced populations based in camps. Reported interventions targeted family planning, HIV/STIs, gender-based violence and general SRH. Most interventions were delivered in hospitals and clinics by doctors and nurses. Delivery barriers included security, population movement and lack of skilled health staff. Multistakeholder collaboration, community engagement and use of community and outreach workers were delivery facilitators. Reporting of intervention coverage or effectiveness data was limited.

**Discussion:**

There is limited relevant literature on adolescents or out-of-camp populations and few publications reported on the use of existing guidance such as the Minimal Initial Services Package. More interventions for gender-based violence were reported in the grey than the indexed literature, suggesting limited formal research in this area. Engaging affected communities and using community-based sites and personnel are important, but more research is needed on how best to reach underserved populations and to implement community-based approaches.

**PROSPERO registration number:**

CRD42019125221.

**Key questions**

**What is already known?**

- Conflict-affected women have additional, specific sexual and reproductive health (SRH) needs as a consequence of their increased vulnerability in such settings, including the higher risk of infectious diseases and higher risk of experiencing gender-based violence.
- Despite the availability of relevant guidance and the findings of previous systematic reviews evaluating the quality, utilisation and effectiveness of available SRH interventions in humanitarian settings, there is still a lack of evidence on the most effective delivery strategies for SRH interventions in armed conflict settings, especially where access to care might be even more limited than in other humanitarian crises.

**Key questions**

**What are the new findings?**

- Skilled health professionals such as doctors and nurses delivered all types of SRH interventions reported in the literature, but the use of community health workers (CHWs) and volunteer community members to deliver a range community-based SRH interventions was also reported.
- Hospitals or fixed clinic settings were reported as delivery sites for all intervention types, but outreach and community-based sites such as health posts, mobile clinics, homes and schools were also reported to be used for some interventions.
- Security constraints and frequent population movement due to conflict were frequently reported barriers to intervention delivery, along with lack of resources, including a lack of skilled health professionals, and social norms that affect the acceptability of SRH. Reported facilitators of intervention delivery included collaboration with local non-governmental organisations, developing and ensuring culturally appropriate interventions and involving community members and influential civic leaders to promote interventions and CHWs to deliver them.
- Most of the literature reports on SRH intervention delivery to camp-based populations, and there is very little documentation of intervention delivery focusing on adolescents, or the delivery of less common SRH interventions such as those for reproductive cancers, which may be important in protracted conflict settings especially.

**What do the new findings imply?**

- A wider range of SRH interventions needs to be provided for a wider range of conflict-affected and displaced populations, with delivery strategies tailored to these settings. While it is still necessary even in conflict settings for skilled health personnel to deliver certain SRH interventions in hospitals and clinics, it is possible, and likely essential, to also engage community-based personnel and places for intervention delivery in order to reach those most in need, many of whom are also the most hard to reach.
- The humanitarian health community, including both practitioners and researchers, need to further strengthen the evidence base on which more specific and actionable guidance on effective SRH intervention delivery strategies in conflict settings can be developed.

## Introduction

Twenty-five years after the 1994 International Conference on Population and Development (ICPD), the fight for the sexual and reproductive health and rights (SRHR) of women continues. The ICPD Programme of Action acknowledged that reproductive health is related to human rights as well as development, and it therefore emphasised women’s rights to access sexual and reproductive health (SRH) services such as family planning, antenatal and delivery care, and safe abortion where legal.^1^ Multiple other global commitments to SRH have been made since the ICPD, the latest being the Sustainable Development Goals (SDGs). Universal access to SRHR by 2030 is included in both SDG 3 on good health and well-being and SDG 5 on gender equality.^2^

Despite these commitments, each year there are still millions of women with an unmet need for modern contraception and safe abortions and men and women without treatment for curable STIs.^3^ Adolescent-specific programming is still not prioritised, and there are other emerging priorities such as the rise of reproductive cancers. These are urgent unmet needs in development as well as humanitarian settings. During the last few decades, the world has experienced an increasing number of conflicts and other humanitarian crises. As of 2018, there were 70.8 million forcibly displaced people worldwide as a result of conflict or persecution, the majority being women and children.^4^ Within conflict-affected settings, aside from the insecurity and displacement that make accessing healthcare more difficult, women have additional specific SRH needs as a consequence of the higher risk of infectious diseases in such settings due to increased vulnerability, and a higher risk of experiencing gender-based violence (GBV).^5 6^

A range of guidance is currently available for humanitarian health response and the delivery of health interventions in conflict and other humanitarian settings. Two key documents include the *Sphere Handbook* and the *Inter-Agency Field Manual (IAFM) on Reproductive Health in Humanitarian Settings* (2018). The *Sphere Handbook* depicts the minimum standards in humanitarian response and contains key specific and comprehensive actions for crisis settings that organisations should undertake. Its chapter on SRH includes sections on general SRH, maternal and newborn care, GBV and HIV management.^7^ The *IAFM on Reproductive Health in Humanitarian Settings* is the authoritative source for SRH in crises, developed by the Inter-Agency Working Group on Reproductive Health in Crises.^8^ The main component of the IAFM is the Minimum Initial Service Package (MISP), which outlines a set of objectives and corresponding priority activities to be undertaken at the onset of a crisis (within 48 hours whenever possible), including the provision of adolescent SRH services, contraception and maternal and newborn health interventions, as well as the prevention of GBV and of HIV and sexually transmitted infections (STIs).^8^

Despite the availability of relevant guidance, there is still a lack of consensus on how best to deliver SRH services in these settings and a need for more scientific evidence on delivery strategies to adequately and effectively meet SRH needs in humanitarian crises. Previous systematic reviews have evaluated the quality, utilisation and effectiveness of available SRH interventions in humanitarian settings,^9–11^ but there is still a lack of evidence on the most effective delivery strategies for SRH interventions in armed conflict settings, especially where access to care might be even more limited than in other humanitarian crises. This review aimed to systematically synthesise the global indexed and grey literature on the delivery of SRH interventions in conflict settings. By examining the delivery platforms, sites and personnel used, and by identifying gaps in the literature, the findings of this review can help to identify potential priorities for action and inform the development of future guidance.

## Methods

### Literature search

We undertook a systematic search of relevant indexed literature published from 1 January 1990 to 31 March 2018 in MEDLINE, Embase, CINAHL and PsycINFO databases using Ovid and EBSCO interfaces. We excluded indexed literature published before 1990 a priori in order to capture as much of the most contemporarily relevant literature as we could feasibly review. We used search terms related to three concepts: (A) conflict; (B) women and children; and (C) SRH. Conflict-related terms included: war, crisis, refugees, internally displaced persons (IDby NGOs or UN agencies, sometimes through the existingPs) and others. Population-related terms included: women, children, pregnant, adolescents, newborns and others. SRH-related terms included: HIV, STIs, GBV and family planning and others. The full MEDLINE search syntax is provided in online supplementary appendix A. Relevant studies from key systematic reviews conducted in the field of the humanitarian health were also screened for potentially relevant publications.

10.1136/bmjgh-2019-002206.supp1 Supplementary data

For grey literature, we searched the websites of 10 major humanitarian agencies and organisations who are actively involved in responding to or researching conflict situations for reports on the implementation of health interventions among women and children. These websites included: Emergency Nutrition Network, International Committee of the Red Cross, International Rescue Committee, Médecins Sans Frontières, Save the Children, United Nations Population Fund, United Nations High Commissioner for Refugees, UNICEF, Women’s Refugee Commission and World Vision. We used broad terms for conflict and health interventions tailored to the search functionality of each website. Given the large volume of potentially relevant grey literature to consider, only documents published from 1 January 2013 to 30 November 2018 were reviewed.

### Eligibility criteria

Our eligibility criteria limited included publications to those reporting on populations affected by conflict in low-income and middle-income countries, as classified by the World Bank.^12^ Included publications must have described an SRH intervention targeting or including neonates, children, adolescents or women of reproductive age, and being delivered during or within 5 years of cessation of a conflict. In order to identify the most informative resources from the large volume of grey literature available, the same inclusion criteria set for indexed literature was applied, with the additional requirement of explicit reporting on the delivery site and personnel for each intervention.

Non-English publications; case reports of single patients; studies reporting on military personnel, on refugee populations bound for a high-income country or on surgical techniques; and pure economic or mathematical modelling studies were excluded from our review. Other exclusion criteria included systematic and literature reviews, meta-analyses, editorials, commentaries, first-person narratives, newspaper and magazine articles, opinion pieces, guidelines and studies where no specific health intervention was described (eg, prevalence studies).

### Data extraction and analysis

All identified indexed records were downloaded into EndNote software, and duplicates were removed. Unique records were then added into Covidence software for screening. Titles and abstracts were screened for relevance by two reviewers independently, with any discrepancies resolved through discussion or by a third reviewer if necessary. The full texts of all potentially relevant publications were reviewed by a single reviewer to determine their eligibility for inclusion in this review, with specific reasons for exclusion noted at this stage. We applied the same approach to the grey literature, with two reviewers screening the title of each retrieved publication for relevance, and a single reviewer then assessing the full text of each potentially relevant publication for eligibility.

We extracted relevant qualitative and quantitative information from all included publications using a customised form on the REDCap platform.^13^ Key variables included setting, population characteristics, intervention components and delivery characteristics, reported delivery barriers and facilitators and any available quantitative data on intervention coverage and effectiveness. Two reviewers extracted and entered information independently; any discrepancies were resolved via discussion or by a third reviewer.

We generated descriptive statistics to summarise key characteristics of the reported populations and interventions, including population displacement status and intervention delivery characteristics, and we narratively synthesised factors impeding or facilitating the delivery of interventions. We tabulated retrieved quantitative data on intervention coverage and effectiveness; given the heterogeneity in the settings, populations and interventions captured in the included publications, we did not undertake meta-analysis.

## Results

### Characteristics of the literature

**Figure 1:**
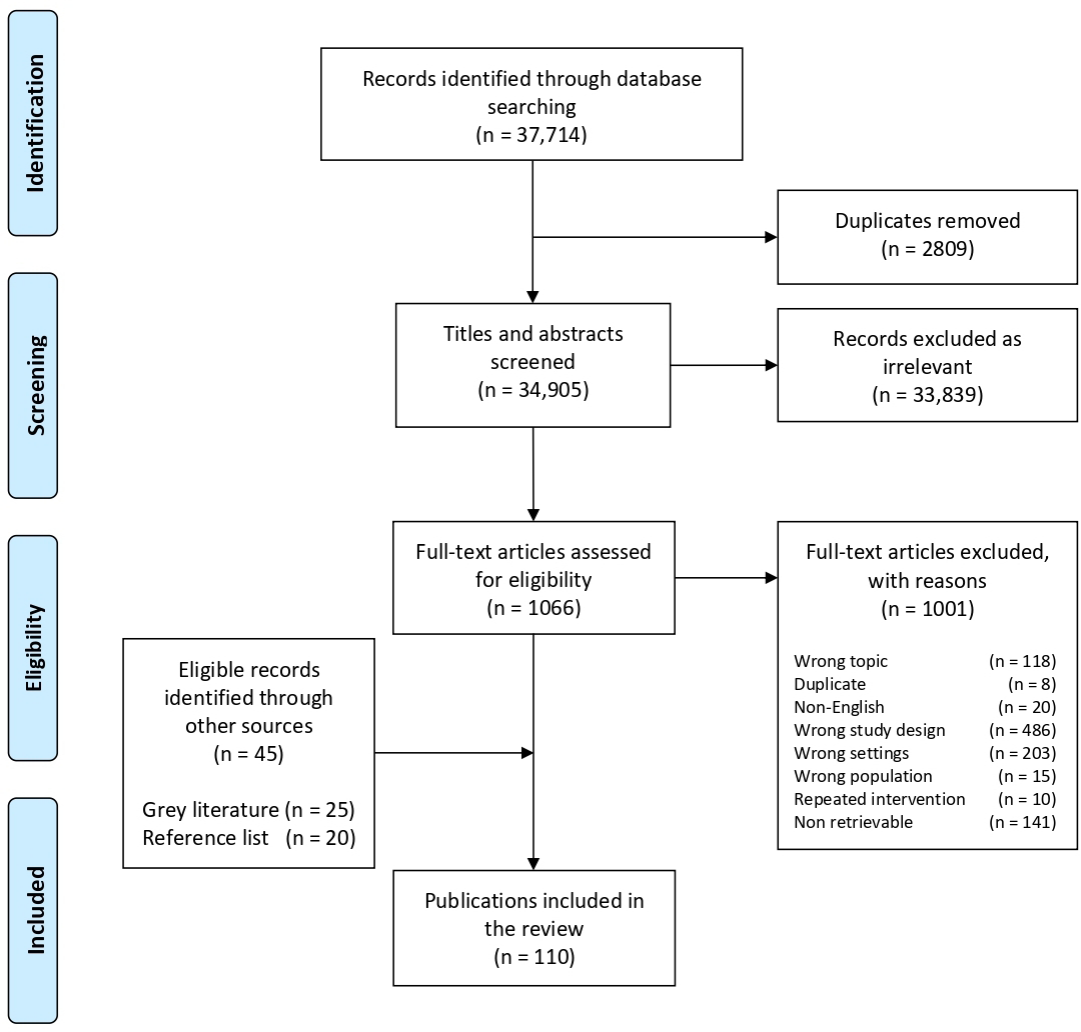
Preferred Reporting Items for Systematic Reviews and Meta-Analyses (PRISMA) flow diagram: publication selectionprocess for systematic review on the delivery of sexual and reproductive health interventions to women and children in conflictsettings.

**Figure 2:**
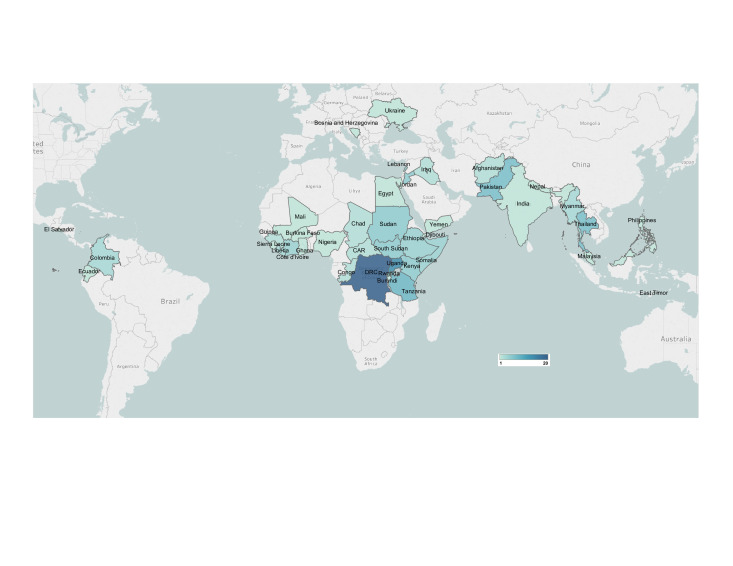
Geographic distribution of included publications.

Our database search retrieved 37 714 indexed publications, of which 65 met our review eligibility criteria. An additional 20 eligible publications were identified from the reference lists of other relevant systematic reviews, and a further 25 eligible publications were identified through our grey literature search and screening (figure 1). The characteristics of all included studies are presented in online supplementary appendix Bonline supplementary file 1. Of the 110 total publications included in our review, the majority were based in sub-Saharan Africa, followed by East Asia and the Pacific region and the Middle East and North Africa region(figure 2). Nearly half of the interventions reported in the literature were delivered to refugees (44%), about one-third were delivered to IDPs (31%) and about 22% were delivered to non-displaced but conflict-affected populations (table 1). Of the publications reporting on interventions delivered to refugees or IDPs, more reported on camp-based populations (73%) than on out-of-camp populations (46%). Most interventions were delivered by NGOs or UN agencies, sometimes through the existing health system and other times in parallel (online supplementary file 2).

10.1136/bmjgh-2019-002206.supp2 Supplementary data

**Table 1:** Characteristics of included publications (n=110) and included interventions (n=331)

Study and population characteristics	
Geographic region*	**n**
East Asia and the Pacific	10
Europe and Central Asia	2
Latin America and the Caribbean	5
Middle East and North Africa	12
South Asia	9
Sub-Saharan Africa	83
Publication type	**n**
Non-research report	54
Mixed methods	4
Observational study	42
Qualitative study	7
Quasiexperimental study	1
Randomised controlled trial	2
Displacement status of beneficiary population*	**n**
Refugees	49
IDPs	49
Non-displaced	25
Returning refugees	3
Host	11
Unreported	14
Setting of displaced populations**†**	**n**
Camp	34
Dispersed	11
Mixed	33
Unreported	10
**Intervention delivery characteristics**	
Target population type*	**n**
All/general population	65
Women of reproductive age	176
Adolescents (10–19 years)	16
Implementation platform*	**n**
Existing health system	110
Faith-based system	9
Informal governance	9
NGO/UN agencies	304
Militaryplatform	3
Researchplatform	31

*Publications can be included in more than one category.

†Only reflects publications that reported on displaced populations (refugees, IDPS or returning refugees).

IDP, internally displaced person; NGO, non-governmental organisation.

**Figure 3:**
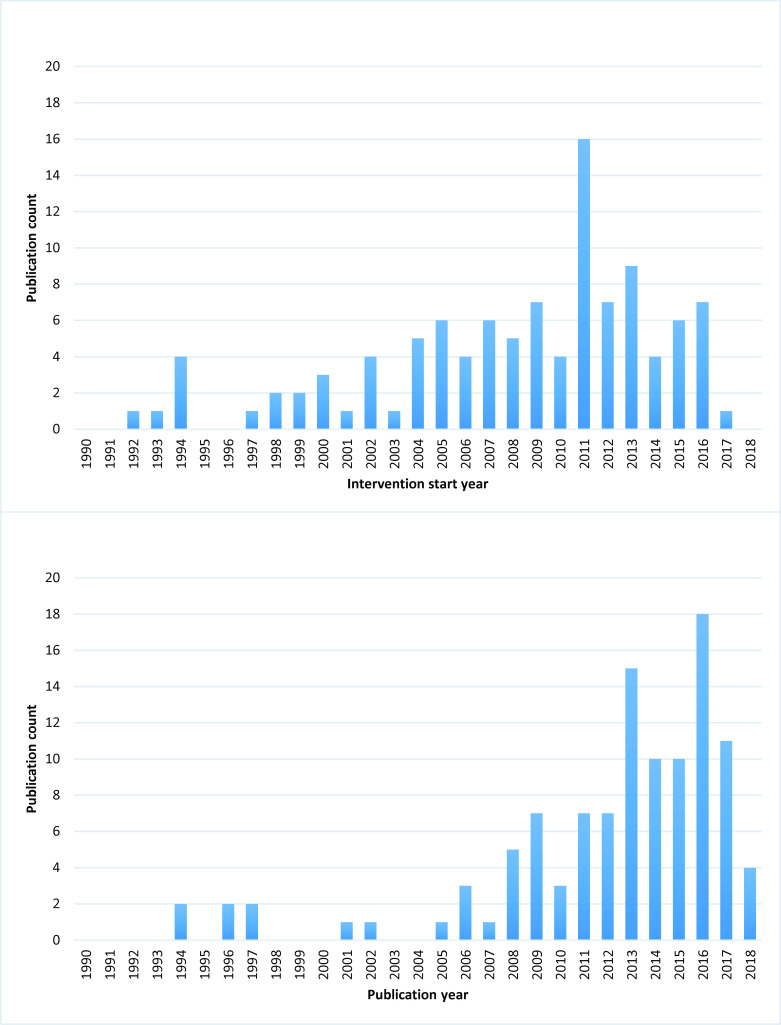
Publication count by publication year and intervention start year

Most of the included literature was published from 2006 onwards, with no more than two publications captured each year before then and none at all before 1994 (figure 3). There were peaks in publications between 2008 and 2009 and again in 2015 and 2017. The reported interventions all started between 1992 and 2017. The increase in interventions delivered between 1994 and 2005 reflects an increase in publications reported on conflict in the Eastern African region, mainly in Rwanda and in the Democratic Republic of Congo (DRC). The later peak in delivered interventions starting in 2011 reflects publications reporting on the ongoing conflict in DRC as well as the increased instability in the Middle East. About half of the included publications were non-research reports (49%), and the remaining were reports of research using mostly observational designs (38%), very few randomised controlled trials (RCTs) (2%) and including a number of qualitative or mixed methods studies (11%).

**Figure 4:**
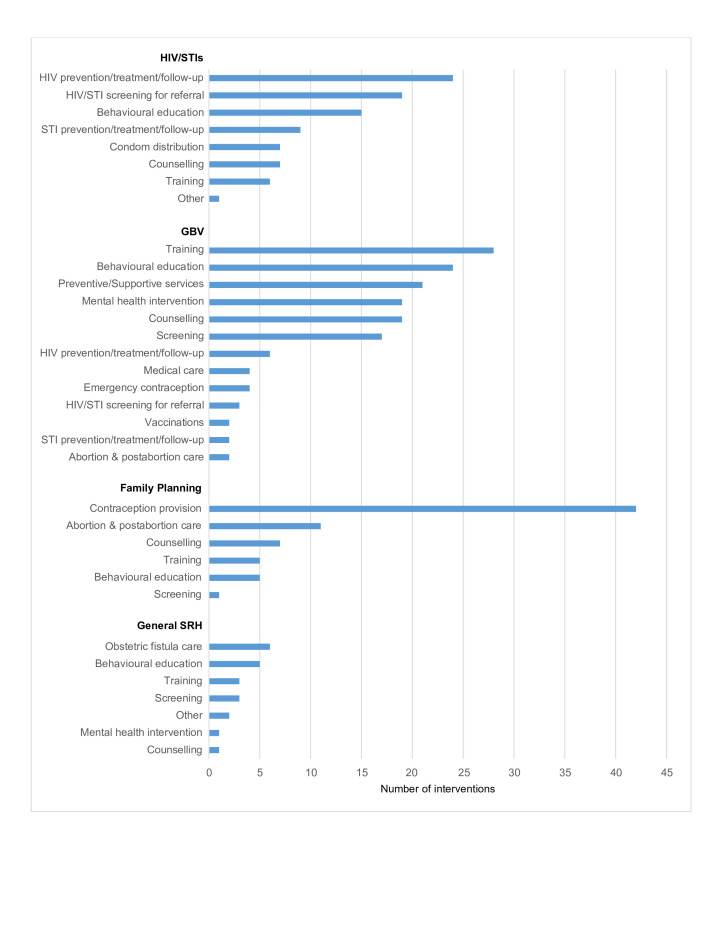
SRH interventions delivered to women of reproductive age. GBV, gender-based violence; SRH, sexual and reproductive health.

Most of the delivered interventions reported in the literature targeted the main components of SRH including HIV and STIs, GBV and family planning, as well as general SRH (figure 4). For HIV and STIs, the most commonly reported interventions delivered were HIV prevention and treatment activities and screening services, followed by behavioural education activities for HIV/STIs and condom distribution. Prevention activities targeting only STIs were not as common as those targeting HIV. Training of healthcare providers was the most frequently reported GBV-focused intervention delivered, followed by the delivery of behavioural educational activities such as distribution of leaflets, drama performances and film showings. The delivery of preventive and supportive services for GBV victims and women at-risk such as the provision of safe spaces, cash transfers and the distribution of hygiene kits was also reported relatively frequently, as well as the delivery of mental health interventions such as psychosocial support, cognitive processing therapy and access to social support groups. Screening interventions to identify victims of GBV were also reported relatively frequently. Regarding the delivery of family planning services, the most commonly reported intervention was contraception provision, with short-acting methods such as the contraceptive pill, injections and condoms as the most commonly reported methods provided. In the publications that mentioned the provision of long-acting reversible contraceptives (LARCs), intrauterine devices (IUDs) were more commonly reported than implants. Very few publications reported on the provision of permanent methods such as sterilisation or vasectomies. The delivery of safe abortion care or postabortion care were the next most commonly reported family planning interventions. There was very limited literature on the provision of safe abortion, however with most of the interventions identified in this category focusing on the delivery of postabortion services and counselling. A few publications reported on the delivery of general SRH interventions, without targeting a specific aspect. These were mostly behavioural educational activities.

**Figure 5:**
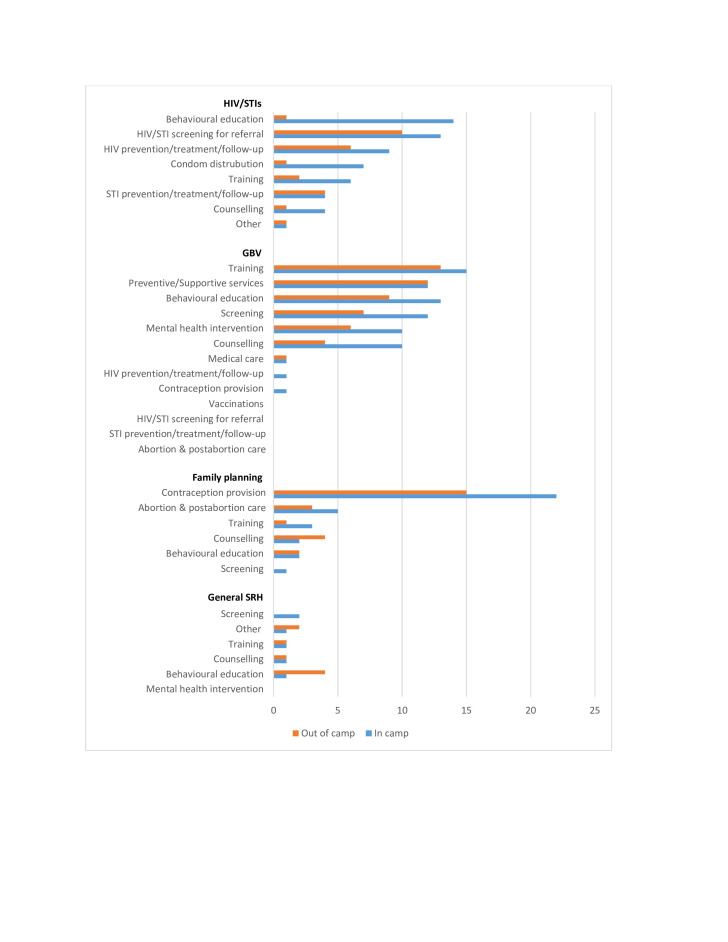
SRH interventions delivered to displaced women of reproductive age living in and outside of camps. SRH, sexual and reproductive health.

Comparing displaced women living in and outside of camps, most of the same interventions were delivered in both settings, but the relative frequency with which they were delivered was different(figure 5). For HIV and STIs, there were more behavioural education activities reported in camp settings compared with out-of-camp settings, mostly community awareness-raising activities. Condom distribution activities were also reported relatively more frequently in camps. There were no stark differences in the reported delivery of GBV-related interventions between settings, although more interventions appear to have been delivered within camps; the same applies to family planning interventions. For general SRH interventions, there were more behavioural interventions reported outside of camps compared with camp settings, while screening interventions were only delivered in camps.

Adolescent interventions reported were mostly on sexual health education, with a focus on HIV and GBV. A few publications also reported on the psychosocial support for adolescent GBV victims.

### Delivery characteristics of reported interventions

**Figure 6:**
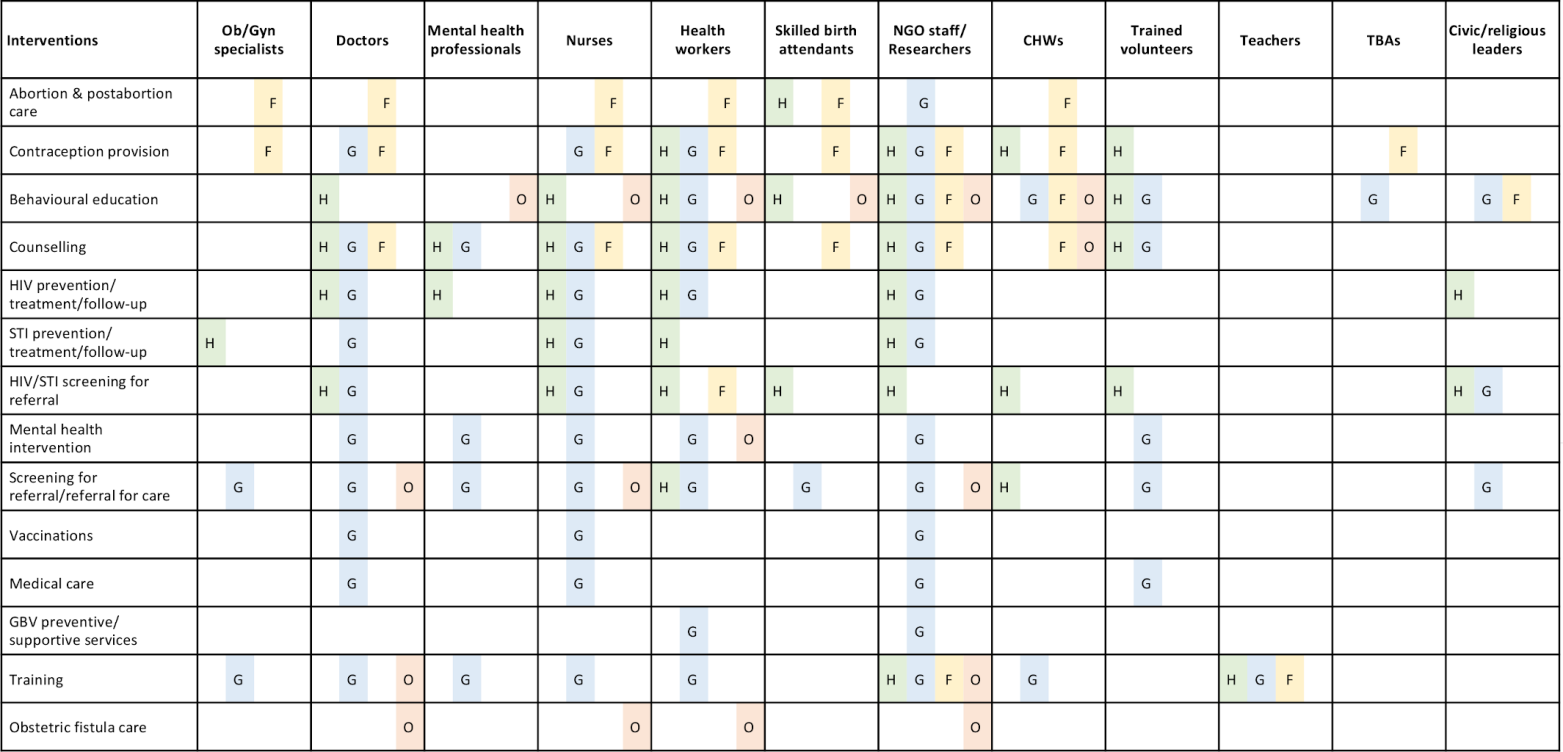
Reported SRH interventions delivered to women of reproductive age, by delivery personnel and SRH domain. CHWs, community health workers; F, family planning (including abortion); G, gender-based violence; GBV, gender-based violence; H, HIV/STIs; O, general SRH; SRH, sexual and reproductive health; TBAs, traditional birth attendants.

Figure 6 maps delivery personnel to delivered interventions, broken down further by the main components or domains of SRH that each intervention was targeting: HIV/STIs, GBV, family planning or general SRH. Among the reported personnel used to deliver SRH interventions, doctors, nurses, health workers and NGO staff or researchers were the most commonly reported, across nearly all types of interventions. Behavioural education, counselling and screening were reported to also have been delivered by trained volunteers. Mental health professionals such as psychologists and psychiatrists were involved in the delivery of GBV-related interventions such as counselling, mental health interventions and the training of other service delivery personnel such as psychosocial assistants. Other commonly reported personnel for GBV-related interventions included social workers, counsellors and psychosocial assistants. Members of the community such as trained volunteers or civic leaders were reported to be involved in the delivery of behavioural education activities, condom distribution, referral for care and some HIV/STI prevention interventions. Community health workers (CHWs) were reported to deliver a range of interventions such as behavioural education, counselling on family planning, screening and referral for care, contraception provision, as well as training of traditional birth attendants (TBAs) on GBV recognition and care for survivors.

**Figure 7:**
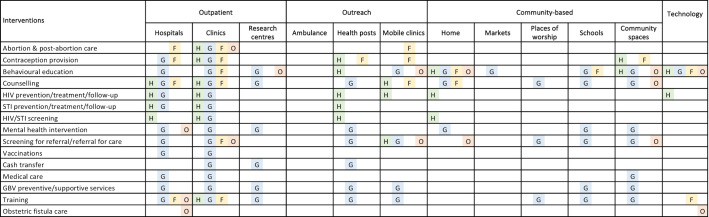
Reported SRH interventions delivered to women of reproductive age, by delivery sites and SRH domain. F, family planning (including abortion); G, gender-based violence; GBV, gender-based violence; H, HIV/STIs; O, general reproductive health; SRH, sexual and reproductive health.

Nearly all SRH interventions were reported to be delivered in both hospitals and clinics (figure 7). Research centres or NGO offices were reported as the delivery site for certain interventions such as behavioural education, counselling, cash transfers and some GBV care. Examples of an outreach approach using delivery sites such as temporary health posts and mobile clinics were also reported for nearly all SRH interventions. Community-based delivery sites included homes, markets, places of worship, schools and communal spaces. These were used in the delivery of a range of interventions such as behavioural education activities, counselling, HIV prevention activities and for training TBAs and community-based volunteers on the immediate care of GBV and to make referrals. Some activities such as community mobilisation and HIV prevention or follow-up care were delivered through communications technology, such as broadcast radio messages or via a telephone hotline for patients with HIV.

Adolescent specific interventions were mostly delivered by NGO staff and in schools or communal spaces, with a few delivered in clinics.

### Intervention coverage and effectiveness

All retrieved quantitative data are presented in online supplementary appendix C. Only 20 (18%) of all publications reported on intervention coverage, and only 8 (7%) reported on intervention effectiveness. The majority of intervention coverage estimates reported on the contraceptive prevalence rate, ranging from 1.7% to 39.6%.^14 15^ Reported coverage of uptake of contraception varied by region. In a study on the Supporting Access to Family Planning and Post-Abortion Care (SAFPAC) initiative in five countries, uptake of LARCs ranged from 1% in Djibouti to 78% in the DRC.^16^ Implants were the most commonly accepted modern method in Chad and the DRC, compared with intrauterine devices (IUDs) or any other modern method. This was different in Pakistan, Mali and Djibouti, where most new users chose another modern method instead of an implant or an IUD.^16^

In the provision of emergency contraception (EC) after sexual assault, there is some indication that coverage may differ when provided by NGO staff compared with doctors or nurses. Only about 50% of those eligible received EC when it was provided by NGO staff in one study^17^ compared with about 90% when it was offered by doctors or nurses in another study.^18^ Other publications reported on the coverage of family planning counselling interventions during postabortion care ranging from 35%^19^ to 98%,^20^ and on GBV interventions such as access to post-exposure prophylaxis ranging from 67%^21^ to 78%,^17^ but coverage patterns by delivery characteristic were not discernible.

Among the effectiveness outcomes reported, some quantified the uptake of contraception after receiving home-based counselling on family planning^22^ or when comparing villages that had a CHW to those that did not^23^; in both cases, the odds of using a modern method of contraception were increased. Bass *et al*^24^ showed a reduction of mean scores of depression and anxiety in women who had experienced sexual violence and who had received individual support and cognitive processing therapy. There were no noticeable differences in intervention effectiveness found between in-camp and out-of-camp populations, but one study by Kim *et al*^25^ identified a higher uptake of voluntary counseling and testing (VCT) services after receiving vouchers for free VCT services in IDP women compared with non-displaced women in surrounding communities.

### Barriers to and facilitators of interventions delivery

Multiple barriers to delivering SRH interventions in conflict settings were reported in the literature; key barriers and examples are presented in table 2. Insecurity as a consequence of ongoing and, in some cases, protracted conflict was cited as an important impediment to intervention delivery in many reports, affecting both logistics and the availability of resources, including commodities and supplies. The lack of sufficient training and availability of appropriate health workers was another common delivery barrier reported, limiting coverage of LARCs, for example. Reported barriers to accessing SRH interventions includedwomen’s restricted mobility as a consequence of displacement due to conflict. Limited personal resources as a result of conflict-induced constraints on income generation was another reported barrier, making the direct and indirect financial costs of accessing services prohibitive in some cases. The social acceptability of the interventions being provided or offeredwas also reported to affect women’s access to SRH interventions, with community social norms and stigma being especially important barriers in some cases.

**Table 2:** Reported barriers to and facilitators of the delivery of SRH interventions

Barriers	
Security	Being in an insecure environment was often mentioned as a hindrance to the delivery of interventions. Health facilities are destroyed, patients are unable to access clinics or clinics are understaffed.
Logistics	Damage to the infrastructure resulting from conflict impeded the operational capacity of healthcare services, difficulties securing transport (fuel and cars) especially when camps are far.
Lack of funding	Limited funding was also noted as a barrier, for example, for family planning programming.
Lack of resources	Shortages of supplies/resources (medicine and diagnostic tests) during conflict were also noted as barriers. In a study by von Roenne *et al* 28, it was noted that district health services were reluctant to provide contraceptives and STI drugs to a local NGO providing services to refugees due to a delay in reimbursement of services delivered to refugees by UNCHR.
Population movement	The continuous population movement limits both delivery and access to health services.
Staff affected by conflict/not buying in	Health services were also limited as staff are also affected by displacement and security concerns. Health workers did not see some health interventions as important.
Lack of skilled/trained health workers	The limited training of health workers was a major barrier in the delivery of interventions such as contraception provision or HIV management. This barrier was noted mostly when it came to providing contraceptives, such as LARCs. In one study, the limited availability of male medical staff was also noted as a barrier for male victims seeking care for sexual assault.^31^
Limited services	Conflict reduces the range of available services. Other factors that were noted to affect interventions such as community mobilisation were poor network coverage/phone charging facilities. Prolonged conflict was also noted as a barrier, as services and support tend to diminish the longer a conflict goes on.^32^
Limited movement for the women/cost barriers	Conflict reduces means of generating income, especially during displacement. Therefore, the cost of getting health services might be weighed against other priorities. In some instances, subsidisation for health services by UNHCR was still not enough.^33^
Social norms/stigma	This was noted as a barrier for both patients as well as healthcare workers. For example, for HIV management, as there is always a lot of stigma associated with it, healthcare workers may not offer all available services or see it as a priority,^34^ while patients may not seek care. Same barrier was apparent for family planning provision (West, 2016). Refugees may also be stigmatised by their hosts.^32^
**Facilitators**	
Collaboration	Multistakeholder collaboration between international NGOs, the Ministry of Health and existing district health offices/public sector were noted as facilitators. Working with local NGOs was also a facilitator as they are already connected to the community
Availability of funding/resources	Having adequate funding allowed for more resources. In one example, the provision of portable CD4 machines by the UNHCR improved treatment quality.^35^
Early preparation	Having a contingency plan for times of disruption and being able to rapidly respond to a conflict were also noted as facilitators, especially for interventions that suffer if disrupted such as antiretroviral therapy (ART) provision.^36 37^
Use of existing infrastructure	Using the existing infrastructure facilitated the delivery of interventions.^38^ Having a stable government, if the conflict is limited to one region, was shown to be a facilitator as it may allow for more organised and consistent services.^14^
Improved systems/innovations	Improving systems such as integrating different activities (nutrition, medical and psychosocial) was a facilitator. Using Geographic Information System (GIS) technology with a mobile clinic was effective in delivering SRH services to IDPs.^27^ Creating safe spaces for girls and women within camps allowed for the delivery of family planning, maternal health and assistance to GBV victims.^39^
Staff training	Training improved the skills of health workers and increased motivation. Continuous supervision/refresher training was encouraged. It was also shown that some mental health interventions for GBV can easily be provided if staff receive training.^24 40^ Tran *et al*^41^ introduced the *Sexual and reproductive health Clinical Outreach Refresher Training* (S-CORT) modules, an innovative approach that focuses on training on the clinical services included in the MISP.
CHWs involvement/outreach workers	Community health workers were seen as trusted members of the community and were useful in delivering interventions such as contraception provision and education on GBV. They were also seen as links between patients and the health system for GBV services.^42^
Community engagement/outreach	Engaging the community through activities such as social mobilisation, empowerment and enabling strategies was a very common facilitator especially as it builds trust. Some approaches used were theatre/drama groups,^18^ and radio broadcast messages.^43^ Peer education was also another strategy used to engage the community or in small groups to address issues such as sexual assault.^44^
Culture/context appropriate	Interventions that were specific to the context and the culture were seen to be more beneficial and as effective even for interventions that were are legally restricted such as abortions.^45^ A study by Wayte *et al*^46^ also found it was necessary to modify guidelines to the local context.
Good leadership/civic/religious leader involvement	Meeting with religious and community leaders were important for building trust and for getting permission to initiate certain interventions that may be innovations, such as CHWs delivering injectable contraceptives, 15 or introducing programming for adolescent girls.^39^
Refugee participation	Refugee participation was noted as a facilitator as it provided manpower and community leadership.^47^ Refugee services run by refugees^28^ were shown to be feasible if there is sustained funding and technical assistance.
Male involvement	Interventions that involved both women and men had better outcomes and more reductions in inter-partner violence (IPV).^48^

CHWs, community health workers; IDPs, internally displaced persons; LARCs, long-acting reversible contraceptives; MISP, Minimum Initial Service Package; NGO, non-governmental organisation; SRH, sexual and reproductive health; UNHCR, United Nations High Commissioner for Refugees.

Despite the many barriers faced by SRH service providers, the literature also reported on factors that facilitated the delivery of SRH interventions, making it easier for implementers to reach their targeted populations. These included effective collaboration between NGOs, ministries of health (MoH) and local civil society organisations (CSOs), to take advantage of existing local infrastructure and the well-established community connections local CSOs often have. The use of CHWs or other outreach workers was also reported to facilitate intervention delivery, as these are often trusted members of the community and serve to link community members to the health system. Engaging target communities through community awareness activities and using community members and peer educators to spread positive informational messages about available services and interventions was another commonly reported facilitator. Multiple publications also highlighted the importance of ensuring culturally and context-appropriate interventions to increase acceptability and reach more people. Several publications also reported on the value of being prepared with contingency plans should the changing security situation disrupt planned intervention delivery. For example, in the provision of ART, cross-training staff in different roles or having emergency drug stocks ready were noted as examples of contingency planning.

## Discussion

### Principal findings

Our review captured 110 publications reporting on the delivery of SRH interventions in conflict settings, many of which mapped to the key priority activities outlined in the *Sphere Handbook* and the *IAFM on Reproductive Health in Humanitarian Settings*. These include activities addressing HIV and STIs, GBV, family planning and general reproductive health, delivered most frequently to camp-based displaced populations, but also to refugees and IDPs residing in open settings, as well as to non-displaced populations. Health professionals such as doctors and nurses were reported to have delivered all types of interventions, but multiple publications also reported on the use of CHWs and volunteer community members to deliver community-based interventions such as awareness-raising activities and condom distribution. Hospitals or fixed clinic settings were reported as delivery sites for all intervention types, but outreach and community-based sites such as health posts, mobile clinics, homes and schools were also reported to be used. GBV-related interventions were mostly delivered within fixed clinics, NGO centres and other spaces where victim safety and protection could be prioritised. The majority of outcomes reported were on the coverage of interventions, with a few on effectiveness. Most coverage indicators were on contraception provision, both for family planning, as well EC for victims of sexual violence. Security constraints and frequent population movement due to conflict were two of the most frequently reported barriers to intervention delivery, with lack of resources, including a lack of skilled health professionals, and social norms that affect the acceptability of SRH interventions also reported as important barriers. Conversely, reported facilitators of intervention delivery included collaboration with local NGOs, developing and ensuring culturally appropriate interventions, and involving community members and influential civic leaders to promote interventions and CHWs to deliver them.

### Evidence gaps

Our findings reveal several important gaps in the existing literature on SRH intervention delivery. The majority of publications captured in our review focused on the delivery of interventions to camp-based populations, rather than to displaced populations settled in open or dispersed settings or to non-displaced populations threatened by armed conflict in situ. Not only does the literature (and potentially also actual progamming) largely fail to capture these largest subpopulations of conflict-affected women,^4^ all with SRH programming needs, but the patterns of delivery for interventions targeted at camp-based populations are likely different from those that might or could be applied to other populations, given the improved access and coverage as well as the higher quality of services often available in camps.^26^

In addition to the non-representativeness of the literature with respect to the displacement status and settings of most conflict-affected women of reproductive age, very few publications reported on the delivery of interventions targeted specifically at adolescents. Although older adolescents were included within interventions targeted at women of reproductive age, there were very few reports of interventions that targeted adolescent SRH.

With respect to the range of interventions reported in the literature, few publications referred explicitly to the MISP guidelines. Among those that did, even fewer reported on the specific components of the MISP that were delivered. There were very few publications that referred to safe abortion or postabortion care. There were also no publications that reported on less common SRH problems such as reproductive cancers, which may be of concern in protracted conflicts especially, and only one publication was found on obstetric fistula. While most publications reported on some type of coverage outcome, either at a single time point or as an estimated difference between two time points, few reported estimates of intervention effectiveness in terms of relevant SRH outcomes.

The majority of reports found in the grey literature focused on GBV, and more so than the peer-reviewed papers, suggesting a lack of formal research on GBV in conflict-affected populations. Alternately, in contrast with the indexed literature, there were no grey literature reports focusing on HIV/STIs, unless they were related to GBV or to general SRH where HIV/STIs were also included. There were also very few reports from the grey literature that provided information on coverage or effectiveness outcomes; however, the grey literature reports tended to provide more detailed insight than indexed publications into the barriers and facilitators of delivering interventions.

### Study strengths and weaknesses

This review is, to our knowledge, the first to focus on how SRH interventions are delivered in settings of armed conflict, and it therefore complements previous systematic reviews on SRH intervention utilisation and effectiveness in humanitarian crises. Previous systematic reviews have shown that SRH interventions that work in non-humanitarian settings such as home visits and peer-led educational and counselling, training of lower level healthcare providers, using CHWs to promote SRH services, involving men in IPV interventions and integrating HIV and SRH services are also effective in humanitarian crises. These interventions and others such as the use of mass media campaigns and community-based programming were also noted to be highly accessed by displaced populations. Our review further corroborates these results, finding that the use of CHWs and outreach workers and engaging the community were reported as facilitators of SRH intervention delivery in conflict settings.

Our review also complements previous reviews by examining how those interventions that have already been shown to be effective and highly accessed are actually being delivered in conflict settings, including specific delivery sites and personnel. Furthermore, our synthesis of the delivery barriers and facilitators can also inform the optimisation of delivery strategies.

There are several limitations to our review, however. Since we necessarily limited our grey literature search to a wide but incomplete landscape of selected NGO or agency websites, we may not have captured all delivered interventions and their respective delivery characteristics. Further exclusions of relevant and potentially informative publications likely resulted from restricting our review to only English language publications. Other limitations may stem from the limited information that is usually provided within the published literature from these settings; not all publications provided detailed information on delivery strategies.

### Potential implications of current findings for future research, programming and policy

Community-based interventions led by CHWs and community members have been highlighted in previous reviews as effective for increasing service utilisation,^10^ and our own review documents the use of community approaches for SRH intervention delivery in conflict settings. The training and engagement of community-based personnel to deliver interventions to conflict-affected populations is a promising strategy for SRH interventions, but there is need for more research and guidance on how best to implement such a strategy and for which SRH interventions this is feasible. Many SRH conditions have stigma associated with them, which can affect intervention acceptability among the population as well as among health personnel. Involving community members and their leaders in SRH intervention delivery might better facilitate implementation through their promotion of intervention acceptability and through their input into the context specificity and cultural appropriateness of proposed interventions.

With respect to SRH intervention delivery sites, the outpatient platform was more commonly reported in the included literature, but we also found multiple examples of outreach and community-based platforms. Wider use of these latter platforms may be especially beneficial in conflict settings, given population movements in many such settings, as well as the prospects for increased engagement of local personnel. Home visits have been shown to be effective,^22^ as well as delivering interventions using outreach activities such as mobile clinics.^27^ One reported innovation was the use of mobile clinics guided by GIS technology to track where displaced populations have moved to.^27^

Our review identified information on the delivery of SRH interventions to out-of-camp populations and to adolescents as two key gaps in the literature. There is a need for more research on both of these conflict-affected populations, in order to identify the best way to reach and engage them. Some innovative methods reported, especially to reach young people, include using drama groups, music and dancers.^28 29^ As there was also a lack of intervention effectiveness data in our results, more rigorous research is also needed to evaluate the effectiveness of SRH interventions in conflict settings.

Although the MISP was mentioned a number of times in the literature, there was otherwise little reference to the use of these common guidelines in these settings. Additional qualitative inquiry on whether and how humanitarian health actors draw on existing evidence and global guidance to develop and deliver their SRH programming in conflict settings could help identify priority areas for further research, guidance and guideline development.

## Conclusions

SRH interventions have the potential to reduce mortality and morbidity in conflict settings, but a wider range of SRH components needs to be addressed in a wider range of conflict-affected and displaced populations, with delivery strategies tailored to these settings. While it is still necessary, even in conflict settings, to use doctors and nurses to deliver certain SRH interventions in hospitals and clinics, it is possible, and likely essential, to also engage community-based personnel and places for intervention delivery in order to reach those most in need, many of whom are also the most hard to reach.^30^ The humanitarian health community, including both practitioners and researchers, need to further strengthen the evidence base on which more specific and actionable guidance on effective SRH intervention delivery strategies in conflict settings can be developed.

## References

[1] United Nations Population Fund (UNFPA) Report of the International Conference on population and development, Cairo. New York: United Nations, 1995: 5–13.

[2] United Nations General Assembly Transforming our world: the 2030 agenda for sustainable development, 2015.

[3] Starrs AM, Ezeh AC, Barker G, et al Accelerate progress—sexual and reproductive health and rights for all: report of the Guttmacher– Lancet Commission. The Lancet 2018;391:2642–92. 10.1016/S0140-6736(18)30293-9 29753597

[4] UNHCR Global trends, forced displacement in 2018, 2018.

[5] Plan UK Factsheet: HIV and failing states. STOPAIDS, 2014.

[6] United Nations Population Fund (UNFPA) The state of the world population 2015: shelter from the storm. A transformative agenda for women and girls in a crisis-prone world. New York, 2015: 1–136.

[7] Sphere Association The Sphere Handbook : Humanitarian charter and minimum standards in humanitarian response. fourth ed Geneva, Switzerland: Sphere Association, 2018.

[8] IAWG Inter-Agency field manual on reproductive health in humanitarian settings. New York: Inter-Agency Working Group on Reproductive Health in Crisis, 2018. 26203479

[9] Singh NS, Aryasinghe S, Smith J, et al A long way to go: a systematic review to assess the utilisation of sexual and reproductive health services during humanitarian crises. BMJ Glob Health 2018;3:e000682. 10.1136/bmjgh-2017-000682 PMC5935157 29736272

[10] Singh NS, Smith J, Aryasinghe S, et al Evaluating the effectiveness of sexual and reproductive health services during humanitarian crises: a systematic review. PLoS One 2018;13:e0199300. 10.1371/journal.pone.0199300 29980147 PMC6035047

[11] Blanchet K, Sistenich V, Ramesh A, et al An evidence review of research on health interventions in humanitarian crisis. United Kingdom, London: London School of Hygiene & Tropical Medicine, Harvard School of Public Health, Overseas Development Institute, 2015.

[12] World Bank Country and Lending Groups The world bank group, 2018 Available: https://datahelpdesk.worldbank.org/knowledgebase/articles/906519-world-bank-country-and-lending-groups

[13] Harris PA, Taylor R, Thielke R, et al Research electronic data capture (REDCap)--a metadata-driven methodology and workflow process for providing translational research informatics support. J Biomed Inform 2009;42:377–81. 10.1016/j.jbi.2008.08.010 18929686 PMC2700030

[14] McGinn T, Austin J, Anfinson K, et al Family planning in conflict: results of cross-sectional baseline surveys in three African countries. Confl Health 2011;5:11. 10.1186/1752-1505-5-11 21752241 PMC3162885

[15] Huber D, Saeedi N, Samadi AK Achieving success with family planning in rural Afghanistan. Bull World Health Organ 2010;88:227–31. 10.2471/BLT.08.059410 20428392 PMC2828781

[16] Curry DW, Rattan J, Huang S, et al Delivering high-quality family planning services in crisis-affected settings II: results. Glob Health Sci Pract 2015;3:25–33. 10.9745/GHSP-D-14-00112 25745118 PMC4356273

[17] Tayler-Smith K, Zachariah R, Hinderaker SG, et al Sexual violence in post-conflict Liberia: survivors and their care. Tropical Medicine International Health 2012;17:1356–60. 10.1111/j.1365-3156.2012.03066.x 22882628

[18] Loko Roka J, Van den Bergh R, Au S, et al One size fits all? standardised provision of care for survivors of sexual violence in conflict and post-conflict areas in the Democratic Republic of Congo. PLoS One 2014;9:e111096. 10.1371/journal.pone.0111096 25329482 PMC4203825

[19] Kinaro J, Ali TEM, Schlangen R, et al Unsafe abortion and abortion care in Khartoum, Sudan. Reprod Health Matters 2009;17:71–7. 10.1016/S0968-8080(09)34476-6 19962640

[20] Chukwumalu K, Gallagher MC, Baunach S, et al Uptake of postabortion care services and acceptance of postabortion contraception in Puntland, Somalia. Reprod Health Matters 2017;25:48–57. 10.1080/09688080.2017.1402670 29231790

[21] Malemo Kalisya L, Lussy Justin P, Kimona C, et al Sexual violence toward children and youth in war-torn eastern Democratic Republic of Congo. PLoS One 2011;6:e15911. 10.1371/journal.pone.0015911 21267467 PMC3022750

[22] Adam IF Evidence from cluster surveys on the association between home-based counseling and use of family planning in conflict-affected Darfur. Int J Gynaecol Obstet 2016;133:221–5. 10.1016/j.ijgo.2015.09.023 26873127

[23] Viswanathan K, Hansen PM, Rahman MH, et al Can community health workers increase coverage of reproductive health services? J Epidemiol Community Health 2012;66:894–900. 10.1136/jech-2011-200275 22068027

[24] Bass JK, Annan J, McIvor Murray S, et al Controlled trial of psychotherapy for Congolese survivors of sexual violence. N Engl J Med 2013;368:2182–91. 10.1056/NEJMoa1211853 23738545

[25] Kim AA, Malele F, Kaiser R, et al HIV infection among internally displaced women and women residing in river populations along the Congo river, Democratic Republic of Congo. AIDS Behav 2009;13:914–20. 10.1007/s10461-009-9536-z 19319674

[26] Spiegel PB, Checchi F, Colombo S, et al Health-Care needs of people affected by conflict: future trends and changing frameworks. Lancet 2010;375:341–5. 10.1016/S0140-6736(09)61873-0 20109961

[27] Shaikh MA Nurses' use of global information systems for provision of outreach reproductive health services to internally displaced persons. Prehosp Disaster Med 2008;23:s35–8. 10.1017/S1049023X00024079 18702286

[28] von Roenne A, von Roenne F, Kollie S, et al Reproductive health services for refugees by refugees: an example from guinea. Disasters 2010;34:16–29. 10.1111/j.1467-7717.2009.01112.x 19459901

[29] Benjamin JA Aids prevention for refugees. The case of Rwandans in Tanzania. Aidscaptions 1996;3:4–9. 12347593

[30] Gausman J, Othman A, Amawi A, et al Child marriage in the Arab world. Lancet 2019;394:825–6. 10.1016/S0140-6736(19)31287-5 31498091

[31] Duroch F, McRae M, Grais RF Description and consequences of sexual violence in Ituri Province, Democratic Republic of Congo. BMC Int Health Hum Rights 2011;11:5. 10.1186/1472-698X-11-5 21504596 PMC3108309

[32] Kabakian-Khasholian T, Mourtada R, Bashour H, et al Perspectives of displaced Syrian women and service providers on fertility behaviour and available services in West Bekaa, Lebanon. Reprod Health Matters 2017;25:75–86. 10.1080/09688080.2017.1378532 29120295

[33] Benage M, Greenough PG, Vinck P, et al An assessment of antenatal care among Syrian refugees in Lebanon. Confl Health 2015;9:8. 10.1186/s13031-015-0035-8 25741381 PMC4349304

[34] O'Brien DP, Mills C, Hamel C, et al Universal access: the benefits and challenges in bringing integrated HIV care to isolated and conflict affected populations in the Republic of Congo. Confl Health 2009;3:1–10. 10.1186/1752-1505-3-1 19126240 PMC2626580

[35] Yiweza Tshipala D, Cornier N, Gounongbe M, et al Ensuring continuity of antiretroviral therapy among displaced populations during Ivorian post-election violence, 2011. J Int AIDS Soc 2012:266–7.

[36] Culbert H, Tu D, O'Brien DP, et al Hiv treatment in a conflict setting: outcomes and experiences from Bukavu, Democratic Republic of the Congo. PLoS Med 2007;4:e129. 10.1371/journal.pmed.0040129 17535100 PMC1880839

[37] Goodrich S, Ndege S, Kimaiyo S, et al Delivery of HIV care during the 2007 post-election crisis in Kenya: a case study analyzing the response of the academic model providing access to healthcare (AMPATH) program. Confl Health 2013;7:25. 10.1186/1752-1505-7-25 24289095 PMC4176498

[38] Krause S, Williams H, Onyango MA, et al Reproductive health services for Syrian refugees in Zaatri cAMP and Irbid City, Hashemite Kingdom of Jordan: an evaluation of the minimum initial services package. Confl Health 2015;9:S4. 10.1186/1752-1505-9-S1-S4 25798190 PMC4331816

[39] Tanner S, O'Conner M A safe place to shine: creating opportunities and raising voices of adolescent girls in humanitarian settings, 2017.

[40] Wirtz AL, Glass N, Pham K, et al Comprehensive development and testing of the ASIST-GBV, a screening tool for responding to gender-based violence among women in humanitarian settings. Confl Health 2016;10:7. 10.1186/s13031-016-0071-z 27099617 PMC4837612

[41] Tran NT, Harker K, Yameogo WME, et al Clinical outreach refresher trainings in crisis settings (S-CORT): clinical management of sexual violence survivors and manual vacuum aspiration in Burkina Faso, Nepal, and South Sudan. Reprod Health Matters 2017;25:103–13. 10.1080/09688080.2017.1405678 29254454

[42] Lokuge K, Shah T, Pintaldi G, et al Mental health services for children exposed to armed conflict: Médecins sans Frontières' experience in the Democratic Republic of Congo, Iraq and the occupied Palestinian Territory. Paediatr Int Child Health 2013;33:259–72. 10.1179/2046905513Y.0000000098 24196701 PMC3817578

[43] Tanaka Y, Kunii O, Hatano T, et al Knowledge, attitude, and practice (KAP) of HIV prevention and HIV infection risks among Congolese refugees in Tanzania. Health Place 2008;14:434–52. 10.1016/j.healthplace.2007.07.005 17954034

[44] O'Callaghan P, McMullen J, Shannon C, et al A randomized controlled trial of trauma-focused cognitive al therapy for sexually exploited, war-affected Congolese girls. J Am Acad Child Adolesc Psychiatry 2013;52:359–69. 10.1016/j.jaac.2013.01.013 23582867

[45] Tousaw E, La RK, Arnott G, et al "Without this program, women can lose their lives": migrant women's experiences with the Safe Abortion Referral Programme in Chiang Mai, Thailand. Reprod Health Matters 2017;25:58–68. 10.1080/09688080.2017.1392220 29210341

[46] Wayte K, Zwi AB, Belton S, et al Conflict and development: challenges in responding to sexual and reproductive health needs in Timor-Leste. Reprod Health Matters 2008;16:83–92. 10.1016/S0968-8080(08)31355-X 18513610

[47] Msuya W, Mayaud P, Mkanje R, et al Taking early action in emergencies to reduce the spread of STDs and HIV. Afr Health 1996;18:24. 12347341

[48] Gupta J, Falb KL, Lehmann H, et al Gender norms and economic empowerment intervention to reduce intimate partner violence against women in rural Côte d'Ivoire: a randomized controlled pilot study. BMC Int Health Hum Rights 2013;13:46. 10.1186/1472-698X-13-46 24176132 PMC3816202

