# Supplementary material for: Delivery of sexual and reproductive health interventions in conflict settings: a systematic review
Source: BMJ Glob Health. 2020 Jul 21;5(Suppl 1):e002206. doi: 10.1136/bmjgh-2019-002206 (PMC7375437; doi:10.1136/bmjgh-2019-002206)
Supplement: Supplementary data [file bmjgh-2019-002206supp001.pdf]

Appendices

Appendix A: MEDLINE Search Strategy

Conflict-related terms

1. disasters/ or emergencies/ or mass casualty incidents/

2. disaster victims/

3. ((disaster or disasters or catastrophe or catastrophes) adj5 (environ* or human or manmade or "man made" or nature or natural or weather)).tw,kf.

4. ("mass casualty" or "mass casualties" or "mass fatalities" or "mass fatality").tw,kf.

5. ((crisis or crises) adj5 (environ* or human or manmade or "man made" or nature or natural or weather)).tw,kf.

6. "warfare and armed conflicts"/ or armed conflicts/ or warfare/ or biological warfare/ or bioterrorism/ or chemical warfare/ or chemical terrorism/ or nuclear warfare/ or psychological warfare/ or war crimes/ or ethnic cleansing/ or genocide/ or holocaust/ or war exposure/ or war-related injuries/

7. afghan campaign 2001-/ or gulf war/ or iraq war, 2003-2011/

8. ("afghan campaign" or "armed conflict" or "armed conflicts" or "gulf war" or "iraq war" or "war time" or "wartime").tw,kf.

9. ((armed or zone or political or civil) adj3 (conflict or conflicts or attack or attacks or war or wars or "no fly")).tw,kf.

10. ("war related injuries" or "war related traumas" or "war related injury" or "war related trauma").tw,kf.

11. ("militant group" or "militant groups" or "militant organization" or "militant organizations" or "militant organisation" or "militant organisations").tw,kf.

12. ("biological terrorism" or bioterrorism or biowarfare or "chemical terrorism" or "ethnic cleansing" or "ethnic cleansings" or "gas poisoning" or genocide or holocaust or holocausts or "nuclear terrorism" or "war exposure" or "war exposures").tw,kf.

13. Disaster Medicine/

14. disease outbreaks/

15. Emergency Medical Services/

16. ((emergency or emergencies) adj5 (environ* or human or manmade or "man made" or nature or natural or weather)).tw,kf.

17. Starvation/

18. (famine or famines or starvation or starvations).tw,kf.

19. cyclonic storms/ or droughts/ or floods/ or tornadoes/ or tidal waves/

20. avalanches/ or earthquakes/ or landslides/ or tidal waves/ or tsunamis/ or volcanic eruptions/

21. (avalanche or avalanches or cyclone or cyclones or drought or droughts or earthquake or earthquakes or flood or flooded or flooding or floods or hurricane or hurricanes or landslide or landslides or "land slide" or "land slides" or mudslide or mudslides or "mud slide" or "mud slides" or storm or storms or tornado or tornadoes or tsunami or tsunamis or typhoon or typhoons or "volcanic ash" or "volcanic eruption" or "volcanic eruptions" or "volcanic gases").tw,kf.

22. refugees/

23. (evacuee or evacuees or refugee or refugees or squatter or squatters or transients).tw,kf.

24. relief work/ or rescue work/

25. ((rescue or relief or aid) adj (plan or plans or activity or activities or agency or agencies)).tw,kf.

26. ("aid plan" or "aid work" or "relief plan" or "relief work" or "rescue plan" or "rescue work").tw,kf.

27. ((staff or staffs or worker or workers) adj3 (relief or aid)).tw,kf.

28. (humanitarian assistance or humanitarian assistances or relief work or relief works).tw,kf.

29. (humanitarian adj2 (aid or response or relief or crisis or crises or emergency or emergencies or disaster or disasters)).tw,kf.

30. Altruism/

31. (humanitarianism or altruism).tw,kf.

32. ("displaced children" or "displaced families" or "displaced family" or "displaced individuals" or "displaced internally" or "displaced men" or "displaced people" or "displaced peoples" or "displaced person" or "displaced persons" or "displaced population" or "displaced populations" or "displaced women" or "forced displacement" or "forced displacements" or "internal displaced" or "internal displacement" or "internally displaced" or "population displaced" or "population displacement").tw,kf.

33. (((camp or camps) and displac*) or "protected village*").tw,kf.

34. (victim or victims).tw,kf.

35. rubble.tw,kf.

36. or/1-35

Population of interest

37. adolescent/ or young adult/

38. (adolescence or adolescent or adolescents or teen* or youth or youths or "young adult" or "young adults").tw,kf.

39. Pregnant Women/

40. exp pregnancy/

41. (expectant or expectancy or gravid* or pregnant or pregnancies or pregnancy).tw,kf.

42. ("mother to be" or "mothers to be").tw,kf.

43. (prenatal or "pre natal").mp.

44. (perinatal or "peri natal").mp.

45. ((trimester or trimesters) adj3 (first or second or mid or third or final or "1st" or "2nd" or "3rd")).tw,kf.

46. (midtrimester or midtrimesters or "early placental phase" or "early placental phases").tw,kf.

47. exp Delivery, Obstetric/

48. ((labor or labour) adj5 (birth* or breech or childbirth or childbirths or complicat* or difficult or early or easy or induce* or induction or late or obstetric* or onset or pregnan* or present*)).tw,kf.

49. parturients.tw,kf.

50. (birth or births or childbirth or childbirths or parturition or parturitions).tw,kf.

51. ("abdominal deliveries" or "abdominal delivery" or "c-section" or "c-sections" or caesarean or caesareans or cesarean or cesareans or "postcesarean section" or "postcaesarean section").tw,kf.

52. exp Abortion, Induced/

53. (abortion or abortions or embryotomies or embryotomy or "postconception fertility control").tw,kf.

54. ((pregnancy or pregnancies) adj3 terminat*).tw,kf.

55. "sexually active".tw,kf.

56. child/ or child, preschool/ or infant/ or infant, newborn/ or infant, low birth weight/ or infant, small for gestational age/ or infant, very low birth weight/ or infant, extremely low birth weight/ or infant, postmature/ or infant, premature/ or infant, extremely premature/

57. (infan* or newborn* or "new born*" or neonat* or baby* or babies or toddler* or boy or boys or boyfriend or boyhood or girl* or kid or kids or child* or pediatric* or paediatric* or peadiatric* or prematur* or preterm*).mp. or school*.tw.

58. refugees/

59. (refugee or refugees).tw,kf.

60. or/37-59

61. 36 and 60

Domain specific terms – Sexual, Reproductive, Maternal & Neonatal health

62. Gynecology/
63. (gynaecology or gynecology).tw,kf.
64. Reproductive Health/
65. ("reproductive health" or "sexual health").tw,kf.
66. Sex Education/
67. ("family planning education" or "family planning instructor" or "family planning instructors" or "family planning training" or "sex education" or "sex instruction").tw,kf.
68. "minimum initial service package".tw,kf.
69. Maternal Health/
70. Maternal Welfare/
71. ("maternal health" or "maternal welfare" or "maternal child health" or "maternal child welfare").tw,kf.
72. Obstetrics/
73. obstetric*.tw,kf.
74. Pregnancy/
75. (pregnanc* or "child bearing" or childbearing).tw,kf.
76. Prenatal Care/
77. ("prenatal" or "pre natal" or "antenatal" or "ante natal").tw,kf.
78. Perinatal Care/
79. ("perinatal care" or "peri natal care" or "perinatal care" or "peri natal care").tw,kf.
80. Peripartum Period/
81. ("peripartum* period*" or "perinatal* period*" or "peri natal* period*").tw,kf.
82. Parturition/
83. (birth or childbirth* or parturition* or "safe delivery" or "safely delivered").tw,kf.
84. (antepartum or "ante partum" or intrapartum or "intra partum").tw,kf.

85. Stillbirth/

86. (stillbirth or stillbirths or stillborn or stillborns or "still birth" or "still births" or "still born" or "still borns").tw,kf.

87. (emoc or emonc or cemoc or bemoc).tw,kf.

88. Midwifery/

89. ("birth attendant" or "birth attendants" or midwife or midwives or midwifery).tw,kf.

90. Postnatal Care/

91. Postpartum Period/

92. (postnatal or "post natal" or postpartum or "post partum" or puerperium or puerperal).tw,kf.

93. ("neonatal care" or "newborn care" or "new born care" or "helping babies breathe" or "helping babies survive").tw,kf.

94. ("neonatal health" or "newborn health" or "new born health" or "infant health").tw,kf.

95. Abortion, Spontaneous/

96. miscarriage*.tw,kf.

97. abortion, induced/ or abortion, eugenic/ or abortion, legal/ or abortion, therapeutic/

98. Abortion, Septic/

99. (abortion or abortions or aborted or aborting or abortus).tw,kf.

100. Misoprostol/

101. (misoprostol or cityl or cyprostol or cytolog or cytotec or gastotec or gastrul or glefos or gymiso or hemoprostol or isprelor or misel or misodel or misofar or misoone or misopress or misoprostil or misotrol or mispregnol or mysodelle or topogyne).tw,kf.

102. Mifepristone/

103. (corlux or corluxin or korlym or lunarette or mifegest or mifegyne or mifeprex or mifepristone or pictovir or "38486" or "r38486" or "r-38486" or "ru 38 486" or "ru 38486" or "ru 486" or "ru38486" or "ru-38486" or "ru486" or "ru-486" or "vgx 410" or "vgx 410c" or "vgx410" or "vgx410c" or "zk 98296" or "zk98296" or "zk-98296").tw,kf.

104. Birth Intervals/

105. ("birth interval" or "birth intervals" or "birth spacing" or "birth spacings" or "child spacing" or "child spacings" or "family building" or "family planning" or "pregnancy interval" or "pregnancy intervals").tw,kf.

106. "safe motherhood".tw,kf.

107. contraception/ or coitus interruptus/ or contraception, barrier/ or contraception, postcoital/ or natural family planning methods/

108. ("birth regulation" or "birth control*" or "coitus interruptus" or "fertility control" or contracept* or "conception control" or antifertility or anticonception or "fertility control" or "fertilization inhibition" or "inhibition of fertilization" or "fertilisation inhibition" or "inhibition of fertilisation").tw,kf.

109. exp Contraceptive Devices/

110. ("cervical cap" or "cervical caps" or "cervix cap" or "dalkon shield" or "dana deveice" or "i.u.d." or "intra uterine device*" or "intracervical device" or "intrauterine device*" or "intrauterine devices" or "intravaginal pessaries" or "intravaginal pessary" or "iucd" or "iud" or "iuds" or "Lippes loop" or "marguilies spiral" or "margulie coil" or "progestin implant" or "vagina pessaries" or "vagina pessary" or "vaginal diaphragm" or "vaginal diaphragms" or "vaginal pessaries" or "vaginal pessary" or "vaginal ring" or "vaginal shield" or "vaginal rings" or "vaginal shields" or "vaginal sponge" or "vaginal sponges" or condom or condoms).tw,kf.

111. Contraceptives, Postcoital/

112. ("morning after pill" or "postcoital antifertility agent" or "postcoital pill").tw,kf.

113. (ulipristal or uliprisnil or "cdb 2914" or "cdb2914" or "ella (drug)" or "ella one" or "ellaone" or "esmya" or "hrp 2000" or "hrp2000" or "rti 3021 012" or "rti 3021-012" or "rti3021 012" or "rti3021-012" or "ru 44675" or "ru44675" or "va 2914" or "va2914").tw,kf.

114. Sexual Abstinence/

115. (celibacy or "postpartum abstinence" or "sexual abstinence" or virginity).tw,kf.

116. hiv/ or hiv-1/ or hiv-2/

117. Human immunodeficiency virus/

118. ("acquired immune deficiency syndrome virus" or "acquired immunodeficiency syndrome virus" or "aids associated lentivirus" or "aids associated retrovirus" or "aids associated virus" or "aids related virus" or "aids virus" or "aids viruses" or "hiv" or "human immuno deficiency virus" or "human immunodeficiency virus" or "human immunodeficiency viruses").tw,kf.

119. HIV Infections/

120. ("hiv infection" or "hiv infections" or "hiv seropositivit*" or "htlv iii infections" or "htlv iii lav infections" or "htlv-iii infection" or "htlv-iii infections" or "htlv-iii-lav infection" or "htlv-iii-lav infections" or "htlv iii seroconversion" or "htlv iii seropositivit*").tw,kf.

121. Acquired Immunodeficiency Syndrome/

122. ("aids" or "acquired immune deficiency syndrome*" or "acquired immuno deficiency syndrome*" or "acquired immunodeficiency syndrome*").tw,kf.

123. Infectious Disease Transmission, Vertical/pc

124. (116 or 117 or 118 or 119 or 120 or 121 or 122) and 123

125. (pmtct or "prevention of HIV mother to child transmission" or "prevention of mother to child transmission" or "eliminate mother to child transmission" or "prevention of mother to child HIV transmission").tw,kf.

126. Sexually Transmitted Diseases/

127. ("sexually transmitted disease*" or "sexually transmitted infection*" or "venereal disease*" or "venereal infection" or "venereal infections").tw,kf.

128. sexually transmitted diseases, bacterial/ or chancroid/ or chlamydia infections/ or lymphogranuloma venereum/ or gonorrhea/ or syphilis/

129. ("calymmatobacterium granulomatis infection" or "chancroid" or "chancroids" or chlamidiosis or "chlamydia infection" or "chlamydia infections" or "Donovania granulomatis infection" or "donovanosis" or "frei disease" or "gonococcal infection" or "gonococcal infections" or "gonococcosis" or "gonococcus infection" or "gonorrhea" or "gonorrheas" or "gonorrhoea" or "granuloma inguinale" or "granuloma venereum" or "great pox" or "inguinal lymphogranulomatosis" or "Klebsiella granulomatis infection" or "lues" or "lymphogranuloma inguinale" or "lymphogranuloma venereum" or "lymphogranuloma venerum" or "lymphopathia venerea" or "lymphopathia venereum" or "Neisseria gonorrhoeae infection" or "nicholas favre disease" or "nicolas favre disease" or "syphilis" or "syphilitic disorder" or "venereal lymphogranuloma").tw,kf.

130. sexually transmitted diseases, viral/ or herpes genitalis/ or Condylomata Acuminata/

131. ("anal wart" or "anal warts" or "anogenital wart" or "anogenital warts" or "condyla acuminatum" or "condylatum acuminatum" or "condyloma accuminatum" or "condyloma acuminata" or "condylomata acuminata" or "genital herpes" or "genital wart" or "genital warts" or "herpes genitalis" or "herpes progenitalis" or "herpes simplex genitalis" or "herpes simplex virus genital infection" or "penile wart" or "penile warts" or "perianal wart" or "perianal warts" or "venereal wart" or "venereal warts" or "verruca accuminata" or "vulvar condyloma").tw,kf.

132. Scabies/

133. ("sarcoptic mange" or scabies).tw,kf.

134. Phthirus/

135. ("crab lice" or "crab lices" or "crab louse" or "crab louses" or "Pediculus pubis" or "phthirus" or "Phtirus pubis" or "Pthirus pubis" or "pubic lice" or "pubic louse").tw,kf.

136. Intimate Partner Abuse/ or Spouse abuse/ or Domestic Violence/

137. ("partner abuse" or "partner violence" or "wife abuse" or "spouse abuse" or "spousal abuse" or "domestic violence" or "domestic abuse" or "gender based violence" or "sex* based violence").tw,kf.

138. ((abuse* or assault* or violence) adj2 (woman or women)).tw,kf.

139. Sex offenses/ or Human Trafficking/ or Rape/

140. ("coerced intercourse" or "forced prostitution" or "forced sex" or "human trafficking" or "human traffickings" or rape or "sex trafficking" or "sex traffickings" or "sex* abuse*" or "sex* assault*" or "sex*

crime*" or "sex* offense" or "sex* offenses" or "sex* slave*" or "sexual aggression" or "sexual bullying" or "sexual coercion" or "sexual exploitation*" or "sexual harassment" or "sexual trauma" or "sexual violence").tw,kf.

141. ("physical* abuse*" or "physical* assault*" or "physical violence").tw,kf.

142. fistula/ or vaginal fistula/ or rectovaginal fistula/

143. (fistula or fistulas or "genital trauma" or "genital injury" or "vaginal trauma" or "vaginal injury").tw,kf.

144. Resuscitation/ and exp Infant, Newborn/

145. ((resuscitat* or reanimat*) adj3 (neonat* or newborn * or "new born*")).tw,kf.

146. Infant Mortality/ and exp Infant, Newborn/

147. Perinatal Mortality/

148. ("neonat* mortalities" or "neonat* mortality" or "neonatal survival" or "newborn mortalities" or "newborn mortality" or "new born mortalities" or "new born mortality" or "newborn survival" or "new born survival" or "perinatal death rate" or "perinatal mortalities" or "perinatal mortality" or "postneonat* mortality" or "postneonat* mortalities").tw,kf.

149. Hypoxia/ and exp Infant, Newborn/

150. Asphyxia Neonatorum/

151. ("asphyxia neonatorum" or "birth asphyxia" or "neonatal anoxia" or "neonatal asphyxia" or "neonatal hypoxia" or "neonate asphyxia" or "neonatus hypoxia" or "new born asphyxia" or "newborn asphyxia").tw,kf.

152. (care adj3 (cord or cords or umbilical or "funiculus umbilicalis")).tw,kf.

153. umbilical cord/ and prolapse/

154. (prolapse* adj3 (cord or cords or umbilical or "funiculus umbilicalis")).tw,kf.

155. chlorhexidine/

156. ("ay 5312" or "ay5312" or "boston conditioning lotion" or "compound 10040" or "mk 412a" or "mk412a" or "sebidin a" or bidex or chlorhex or chlorhexidin or chlorhexidine or chlorohex or chlorohexidine or chlorohexydine or clohexidine or lisium or nibitane or nolvasan or nolvascin or novalsan or rotersept or sterilon or tubilicid or tubulicid or umbipro).tw,kf.

157. Neonatal Sepsis/

158. ("neonatal early onset sepses" or "neonatal early onset sepsis" or "neonatal late onset sepses" or "neonatal late onset sepsis" or "neonatal sepses" or "neonatal sepsis" or "neonatal septicaemia" or "neonatal septicemia" or "new born sepses" or "new born sepsis" or "new born septicaemia" or "new born septicemia" or "newborn sepses" or "newborn sepsis" or "newborn septicaemia" or "newborn septicemia").tw,kf.

159. Kangaroo-Mother Care Method/

160. ("kangaroo mother care" or "kangaroo mother method" or "skin to skin").tw,kf.

161. exp meningitis/ and exp Infant, Newborn/

162. ((meningitides or meningitis or "meningeal inflammation" or pachymeningitides or pachymeningitis or "perimeningeal infections") adj3 (neonat* or newborn * or "new born*")).tw,kf.

163. Jaundice, Neonatal/

164. ("bronze baby syndrome" or erythroleukoblastosis or ((jaundice or icterus) adj3 (neonat* or newborn * or "new born*"))).tw,kf.

165. Fetal Growth Retardation/

166. ("congenital hypotrophy" or "fetal growth disorder" or "fetal growth restriction" or "fetal growth retardation" or "fetus growth disorder" or "fetus growth retardation" or "foetal growth restriction" or "foetal growth retardation" or "growth retardation in utero" or "in utero growth retardation" or "intrauterine growth restriction" or "intrauterine growth retardation" or "iugr" or "prenatal growth retardation" or "retarded intrauterine growth").tw,kf.

167. Eclampsia/ or Pre-eclampsia/

168. ("eclamptic toxaemia" or "eclamptic toxemia" or "eclamptogenic toxaemia" or "eclamptogenic toxemia" or "edema proteinuria hypertension gestosis" or "eph complex" or "eph gestosis" or "eph syndrome" or "eph toxemia" or "eph toxemias" or "gestational toxaemia" or "gestational toxemia" or "gestational toxicosis" or "hep syndrome" or "hypertension edema proteinuria gestosis" or "pre eclampsia" or "pre eclamptic toxaemia" or "pre eclamptic toxemia" or "preeclamptic toxaemia" or "preeclamptic toxemia" or "pregnancy toxaemia" or "pregnancy toxaemias" or "pregnancy toxemia" or "pregnancy toxemias" or "pregnancy toxicosis" or "proteinuria edema hypertension gestosis" or "toxaemia gravidum" or "toxemia gravidum" or "toxemia of pregnancies" or "toxemia of pregnancy" or "toxemic pregnancy" or eclampsia or eclampsias or preeclampsia).tw,kf.

169. dystocia/

170. ("abnormal labor" or "abnormal labour" or "delayed labor" or "delayed labour" or "inertia uteri" or "labor obstruction" or "labour obstruction" or "obstructed labor" or "obstructed labour" or "uterus inertia" or dystocia or dystocias).tw,kf.

171. Breech Presentation/

172. (breech adj2 (present* or position*)).tw,kf.

173. Uterine Hemorrhage/

174. ("vagina* haemorrhage" or "vagina* hemorrhage" or "vaginal bleeding").tw,kf.

175. or/62-115,124-174

176. 61 and 175

177. limit 176 to dc=20170530-20180331

178. ("2017 05 30*" or "2017 05 31*" or "2017 06*" or "2017 07*" or "2017 08*" or "2017 09*" or "2017 10*" or "2017 11*" or "2017 12*" or "2018 01*" or "2018 02*" or "2018 03*").dt.

179. 176 and 178

180. 177 or 179

Appendix B. Characteristics of included publications (Full citations are listed below)

Author	Report type	Country	Displacement status	Setting	Target population	Intervention	Delivery platform	Delivery site	Delivery personnel
Adam et al (2015)	Observational study	Sudan	IDPs	Camp	WRA, Pregnant women, Postnatal mothers, Neonates	Counselling, contraception provision, HIV/STI behavioural education	Healthcare system, NGO/UN Agency	Clinics, home	CHWs, doctors, medical assistant, nurses, SBAs
Adam, I.F (2016)	Observational study	Sudan	IDPs	Camp	WRA	Contraception provision and counselling	NGO/UN Agency	Clinics, home	CHWs
Not reported	Non-research	Rwanda	Refugees	Camp	General population	Condom distribution	NGO/UN Agency	NR	Unreported
Balsara et al (2010)	Observational study	Pakistan	Refugees	Camp	Adolescents, Women >20 y	Screening for referral with intent to treat (other than STIs)	NGO/UN Agency	Clinics	Medical Officers
Bannink-Mbazzi et al. (2013)	Observational study	Uganda	IDPs, Not displaced	Camp	Infants (0-18 mo), Pregnant women, general population	HIV prevention, treatment and follow-up care, HIV/STI screening for referral, behavioural education	Healthcare system, NGO/UN Agency	Clinics,Electronic/ print	Health workers
Bass et al. (2013)	Quasi-experimental/ Non-randomized control trial	DRC	NR	N/A	Adolescents, Women >20 y, Pregnant women, Post-natal mothers	Psychosocial support, cognitive processing therapy, training	NGO/UN Agency, Research	Research centres	NGO staff, researchers, psychosocial assistants
Benage et al (2015)	Observational study	Lebanon	Refugees	Dispersed	Pregnant women	Contraception provision	Healthcare system, NGO/UN Agency	Clinics, communal spaces	Health workers
Benjamin et al (1996)	Non-research	Tanzania	Refugees	Camp	Adolescents, General population	Behavioural education, screening for referral, condom distribution, home-based care, social support groups	NGO/UN Agency	Clinics, Home, communal spaces, electronic/print	AIDS community educators (ACEs), counselors, NGO staff, refugee social worker, volunteers, traditional dancers
Bhardwaj et al. (2018)	Observational study	Nepal	NR	N/A	Adolescents, General population	Counselling	NGO/UN Agency	Unreported	Counsellors
Bile et al (2011)	Non-research	Pakistan	IDPs	Camp, Dispersed	Pregnant women	Contraception provision	Healthcare system, NGO/UN Agency	Clinics	CHWs
Bosmans et al (2012)	Non-research	Colombia	IDPs	NR	Adolescents	Sexual health education and promotion	Healthcare system, NGO/UN Agency, Research	Schools	Health workers

Author	Report type	Country	Displacement status	Setting	Target population	Intervention	Delivery platform	Delivery site	Delivery personnel
Casey et al (2006)	Observational study	Sierra Leone	IDPs	Camp, Dispersed	Young people (15-24 years)	Contraception provision, behavioural education	NGO/UN Agency	NR	ARC Health Team
Casey et al (2011)	Non-research	DRC	NR	N/A	WRA	Behavioural education, emergency contraception, HIV post-exposure prophylaxis, training	NGO/UN Agency	Clinics	Health workers
Casey et al (2013)	Observational study	Uganda	IDPs	Camp, Dispersed	WRA	Contraception provision	Healthcare system, NGO/UN Agency	Clinics, mobile clinics	Doctors,Nurses, SBAs
Casey et al (2017)	Observational study	DRC	Not displaced	N/A	WRA	Contraception provision	Healthcare system, NGO/UN Agency	Clinics, hospitals	Trained providers
Cherri et al (2017)	Qualitative study	Lebanon	Refugees	Dispersed	WRA	Contraception provision	NGO/UN Agency	Clinics	Unreported
Chukwumalu et al. (2017)	Observational study	Somalia	IDPs	NR	WRA	Family planning counselling and contraception provision, abortion and post-abortion care, training	Healthcare system, NGO/UN Agency	Clinics, hospitals	CHWs,nurses, skilled birth attendants, Health workers, NGO staff
Cohen, R.A. (2013)	Non-research	Ecuador	Refugees	Dispersed	Women >15 y	Psychotherapeutic activities	Research	Unreported	Researchers, health workers, counsellors, an artist, an anthropologist, and a seamstress.
Culbert et al (2007)	Observational study	DRC	IDPs, Not displaced	NR	General population	HIV treatment, behavioural education, screening for referral, prophylaxis and treatment for opportunistic infections including tuberculosis, counselling, nutritional support	Healthcare system, NGO/UN Agency	Clinics, hospitals	Doctors,Nurses, Trained civilians, Health workers
Curry et al. (2015)	Non-research	Chad, DRC, Djibouti, Mali, Pakistan	IDPs, Hosts, Refugees	Camp	WRA	Abortion and post-abortion care, contraception provision (including condoms)	Healthcare system, NGO/UN Agency	Clinics, hospitals	CHWs, Doctors, Nurses, Health workers
Doumbouya et al (2012)	Non-research	Côte d'Ivoire	Not displaced	N/A	General population	HIV treatment	NGO/UN Agency	Clinics	Medical staff, Counsellors and data managers

Author	Report type	Country	Displacement status	Setting	Target population	Intervention	Delivery platform	Delivery site	Delivery personnel
Duroch et al (2011)	Observational study	DRC	NR	N/A	Children (1-9 years), Adolescents, Women >20 y, Pregnant women, Post-natal mothers, General population	Cmprehensive medical care for victims of sexual violence	NGO/UN Agency	Clinics, hospitals	Unreported
Ehui et al (2015)	Observational study	Côte d'Ivoire	NR	N/A	Children (1-9 years), Adolescents	HIV treatment, contraception provision, antibiotics	Healthcare system	Clinics, hospitals	Health workers,Social workers
Ellman et al (2005)	Non-research	DRC	Not displaced	N/A	General population	HIV treatment	NGO/UN Agency	Clinics	Health workers
Erickson et al (2015)	Observational study	Uganda	IDPs, Refugees	Camp	Adolescents, Women >20 y, Pregnant women, Post-natal mothers, General population	Screening for referral with intent to treat (other than STIs)	NGO/UN Agency	Clinics	NR
Foster et al. 2017	Observational study	Thailand	Refugees	Camp, Dispersed	Pregnant women	Abortion and post-abortion care	NGO/UN Agency	Clinics	Doctors,Health workers,Other
Frljak et al (1997)	Observational study	Bosnia and Herzegovi na	Refugees, Not displaced	Dispersed	Adolescents, Women >20 y, Pregnant women, Post-natal mothers	STI prevention, treatment and follow-up care, abortion and post-abortion care, contraception provision, GBV counselling	NGO/UN Agency	Clinics, mobile clinics	NursesGyneocologis t
Garang et al. (2009)	Observational study	Uganda	IDPs, Not displaced	Camp, Dispersed	Women >15 y, Pregnant women, Post-natal mothers, General population	HIV prevention, treatment and follow-up care	NGO/UN Agency	Hospitals	Doctors,NGO staff,Health workers
Gedeon et al (2015)	Qualitative study	Thailand	IDPs, Refugees	Camp, Dispersed	WRA	Contraception provision	Research	Clinics	Unreported
Goodrich et al. 2013	Non-research	Kenya	IDPs, Not displaced	Camp	General population	HIV prevention, treatment and follow-up care (emergency hotline, prophylaxis for ooportunistic infections, counselling)	Healthcare system, Research	Clinics,Mobile clinics, electronic/print	Doctors, nurses, Health workers, psychosocial support staff, nutritionists, social workers
Gupta et al. (2013)	Randomized control trial	Côte d'Ivoire	NR	N/A	Women >15 y, general population	Counselling (gender dialogue group), Economic empowerment activities (village saving and loans associations)	NGO/UN Agency, Research	Unreported	NGO staff, Researchers

Author	Report type	Country	Displacement status	Setting	Target population	Intervention	Delivery platform	Delivery site	Delivery personnel
Gurman et al . (2014)	Non-research	Liberia, Rwanda, South Sudan, Thailand, Uganda	Refugees	Camp	Adolescents, Women >20 y, Pregnant women, Post-natal mothers, General population	Behavioural education, GBV training	Faith-based system, Informal governance/Civic leadership, NGO/UN Agency	NR	NGO staff, community members.
Hampton T. (2008)	Non-research	Uganda	IDPs	Dispersed	General population	HIV prevention, treatment and follow-up care	NGO/UN Agency	Clinics, home	Health workers
Hemhongsa et al (2008)	Observational study	Thailand	Refugees, Not displaced	Camp	General population	HIV treatment , Counselling	Healthcare system, NGO/UN Agency, Research	Clinics, hospitals	Doctors,Nurses
Huber et al. (2010)	Observational study	Afghanist an	Not displaced	N/A	WRA	Family planning counselling and contraception provision	Healthcare system, NGO/UN Agency	NR	CHWs
Hustache et al. (2009)	Observational study	DRC	NR	N/A	Women >15 y,Pregnant women,Post-natal mothers	Psychological support	Healthcare system, NGO/UN Agency	Home, hospitals	Psychologist, social worker
Iyakaremye et al. (2016)	Qualitative study	Rwanda	Refugees	Camp	Adolescents	GBV education, post-exposure prophylaxis, emergency contraception	NGO/UN Agency	Clinics	Health workers, Trained civilians
Kabakian-Khasholian et al. (2017)	Qualitative study	Lebanon	Refugees	Dispersed	WRA, Pregnant women, post-natal mothers,Neonates	Contraception provision	Healthcare system, NGO/UN Agency	Clinics	NR
Kaiser et al(2006)	Observational study	Sudan	IDPs	NR	WRA, Pregnant women, Post-natal mothers, General population	Counselling, STI screening for referral, antibiotics	Research	Clinics	NR
Kiboneka et al. (2009)	Non-research	Uganda	IDPs	Camp, Dispersed	Women >15 y, General population	HIV prevention, treatment and follow-up care	NGO/UN Agency	Clinics, Home, Mobile clinics	Doctors,NGO staff,Health workers, Civic leader
Kim et al. (2009)	Observational study	DRC	IDPs, Returning refugees, Not displaced	Camp, Dispersed	WRA	Referral vouchers, HIV/STI testing and counselling, STI treatment and follow-up care	Research	Clinics	NR
Kinaro et al. (2009)	Mixed methods study	Sudan	IDPs, Not displaced	Dispersed	WRA, Pregnant women	Abortion and post-abortion care, family planning counselling and contraception provision	Healthcare system, NGO/UN Agency	Hospitals	Doctors

Author	Report type	Country	Displacement status	Setting	Target population	Intervention	Delivery platform	Delivery site	Delivery personnel
Kisindja et al. (2017)	Observational study	DRC	IDPs	Camp	WRA	Behavioural education	NGO/UN Agency	NR	NGO staff, civic leaders
Krause et al. (2015)	Mixed methods study	Jordan	Refugees	Camp, Dispersed	WRA	Minimum Initial Service Package (MISP)	Healthcare system, Research	Clinics, hospitals	Health workers
Lee et al (2008)	Non-research	Philippine s	NR	N/A	WRA, pregnant women	Contraception provision, counselling	Healthcare system, NGO/UN Agency	Hospitals,clinics, health posts	Doctors, nurses, midwives, healthworkers,
Loko Roka (2014)	Observational study	DRC	NR	N/A	WRA, general population	HIV/STI screening, emergency contraception, prophylaxis for STIs and HIV, hepatitis B and tetanus vaccinations, psychological counselling, behavioural education	Healthcare system, NGO/UN Agency	Hospitals, clinics, communal spaces	NGO staff, doctors, nurses, CHWs
Lokuge et al. (2013)	Observational study	DRC	IDPs	Camp, Dispersed	Infants (1-12 mo), Children (1-9 y), Adolescents	Counselling, behavioural education, screening for referral	NGO/UN Agency	Hospitals	Lay counsellors, NGO staff
Malemo Kalisya et al. (2011)	Observational study	DRC	NR	N/A	Infants (1-12 mo), Children (1-9y), Adolescents, Women >20 y, Pregnant women, Post-natal mothers	HIV/STI screening for referral, Post-exposure prophylaxis	NGO/UN Agency	Hospitals	NR
Mankuta et al. (2012)	Observational study	DRC	NR	N/A	Adolescents, Women >20 y, Pregnant women, Post-natal mothers	Training, screening for referral, gynecological reconstructive surgery, PTSD treatment using the EMDR technique (eye movement desensitization and reprocessing)	NGO/UN Agency	Health posts, hospitals	Doctors,Nurses,gyn ecologists, psychologist
Mayaud P. (2001)	Observational study	Tanzania	Refugees, hosts	Camp	General population, pregnant women	Sexual health education and promotion, condom provision, screening for referral, antibiotics, training	NGO/UN Agency	Clinics	Trained civilians, peer educators, healthworkers
Mayaud et al. (1997)	Observational study	Tanzania	Refugees	Camp	Pregnant women,General population	STI screening for referral, antibiotics	NGO/UN Agency	Clinics	Unreported

Author	Report type	Country	Displacement status	Setting	Target population	Intervention	Delivery platform	Delivery site	Delivery personnel
McGinn et al (2006)	Observational study	Guinea	Refugees	Camp	Women >20 y, Pregnant women, Post-natal mothers	Training	NGO/UN Agency, Research	NR	NGO staff, Researchers, teachers
McGinn et al. (2011)	Observational study	DRC, Sudan, Uganda	IDPs, Not displaced	Camp, Dispersed	WRA	Contraception provision, behavioural education	Healthcare system, NGO/UN Agency	Clinics, hospitals	Unreported
Mendelsohn et al. (2014)	Observational study	Malaysia	Refugees, hosts	Dispersed	Women >15 y,Pregnant women,Post-natal mothers,General population	HIV prevention, treatment and follow-up care	Healthcare system, NGO/UN Agency	Hospitals	Unreported
Mitike, G. & Deressa, W. (2009)	Observational study	Ethiopia	Refugees	Camp	General population	Training, FGM behavioural education	Healthcare system, NGO/UN Agency	Clinics	Civic leaders ,CHWs,religious leaders,Health workers
Mogga R. (2017)	Non-research	Uganda	Refugees	Camp	Adolescents, Women >20 y, Pregnant women, Post-natal mothers	GBV screening for referral, cognitive behavioural treatment therapy (CBTT), psychological first aid (PFA), counselling	NGO/UN Agency	NR	Clinical psychiatric officer, clinical psychologist, social workers
Morrison et al. (2000)	Mixed methods study	Thailand	Refugees	Camp	Pregnant women, post natal mothers, Women >15 y	Contraception provision, STI screening for referral, training	Healthcare system, NGO/UN Agency	Clinics, hospitals	Doctors, midwives, NGO staff,TBAs
Msuya et al. (1996)	Non-research	Tanzania	Refugees	Camp	General population	STI screening for referral, antibiotics, behavioural education, condom provision	NGO/UN Agency	Clinics, electronic/print, communal spaces	NGO staff, nurses, SBAs, health behaviour promoters, peer educators
Mukwege, D., Berg, M. (2016)	Non-research	DRC	Not displaced	N/A	Children (1-9 years), Adolescents, Women >20 y, Pregnant women, Post-natal mothers, General population	Counselling, screening for referral (STIs, and other), specialist neuropsychiatric treatment	Healthcare system	Hospitals, mobile clinics, health posts,	Doctors, psychologist, psychosocial workers, police, parajudicial assistants
Nattabi et al. (2011)	Mixed methods study	Uganda	IDPs, Not displaced	Camp, Dispersed	WRA, General population	Family planning counselling and contraception provision	Healthcare system, NGO/UN Agency	Clinics, mobile clinics	Health workers
O'Brien et al (2009)	Non-research	Republic of Congo	NR	N/A	General population	HIV treatment, counselling	Healthcare system, NGO/UN Agency	Clinics, hospitals	Doctors,Nurses,Health workers

Author	Report type	Country	Displacement status	Setting	Target population	Intervention	Delivery platform	Delivery site	Delivery personnel
O'Callaghan et al. (2013)	Randomized control trial	DRC	IDPs, Not displaced	NR	Adolescents	Mental health intervention (trauma focused cognitive behavioural therapy (TF-CBT), hygiene kit distribution	NGO/UN Agency	Schools	NGO staff, social workers
O'Laughlin et al. (2014)	Observational study	Uganda	Refugees, Not displaced	Camp, Dispersed	Women >15 y,Pregnant women,Post-natal mothers,General population	HIV/STI screening for referral	NGO/UN Agency, Research	Clinics,Health posts	NGO staff, Researchers
Palmer et al. (2016)	Qualitative study	South Sudan	IDPs	Camp	WRA	Abortion and post-abortion care, contraception provision	Healthcare system, NGO/UN Agency	Clinics	Health workers
Reid et al. (2008)	Non-research	Kenya	Not displaced	N/A	General population	Call centre with a toll free line	Healthcare system, NGO/UN Agency	Electronic/print	NGO staff
Rodger et al. (2002)	Observational study	India	IDPs	Camp, Dispersed	General population	HIV/STI screening for referral	Healthcare system, NGO/UN Agency	Clinics	Civic leader,CHWs,NGO staff
Rosenberg S., Bakomeza D. (2017)	Non-research	Uganda	Refugees	Camp, Dispersed	Women >15 y,Pregnant women,Post-natal mothers	Training HIV/STIs and GBV, HIV/STI screening for referral and counselling	NGO/UN Agency	Clinics	NGO staff, peer educators
Rowley et al. (2008)	Observational study	Tanzania	Refugees, hosts	Camp, Dispersed	WRA,Pregnant women,Post-natal mothers,General population	HIV/STI screening for referral and counselling, condom provision	NGO/UN Agency	Clinics, pharmacies,	NGO staff, CHWs, Health workers
Rutta et al. (2008)	Observational study	Tanzania	Refugees	Camp	Infants (0-18 mo), pregnant women, post-natal mothers	HIV counselling and testing, HIV prevention, treatment and follow up care (home-based care, nutritional support, infant-feeding counselling) , behavioural education, training	Healthcare system, NGO/UN Agency	Clinics, hospitals	CHWs,Doctors,Nurses, SBAs, Health workers, counsellors
Salami et al. (2010)	Observational study	South Sudan	IDPs, Returning refugees	Camp, Dispersed	General population	HIV prevention, treatment and follow-up care	NGO/UN Agency	Clinics	NR
Shaikh M. A., (2008)	Non-research	Somalia	IDPs	Camp	Women >15 y, pregnant women, post-natal mothers	Screening for referral	NGO/UN Agency	Mobile clinics	Nurses, SBAs

BMJ Publishing Group Limited (BMJ) disclaims all liability and responsibility arising from any reliance placed on this supplemental material which has been supplied by the author(s)

Author	Report type	Country	Displacement status	Setting	Target population	Intervention	Delivery platform	Delivery site	Delivery personnel
Shamomesh et al. (1994)	Observational study	El Salvador	Returning refugees	Dispersed	Women >15 y,Pregnant women,Post-natal mothers	STI screening and treatment	Healthcare system, Research	Clinics	Health workers
Somigliana et al. 2011	Observational study	Uganda	NR	N/A	Neonates, Women (10-49 y), Pregnant women	ART provision, safe delivery care, contraception provision, adolescent SRH services provision	NGO/UN Agency	Clinics,Hospitals, ambulance	Health workers, gynecologists, traditional birth attendants
Tanabe et al. (2013)	Qualitative study	Burma (Myanmar)	IDPs	Dispersed	Adolescents, Women >20 y, Pregnant women, Post-natal mothers	Training, GBV behavioural education	Healthcare system, NGO/UN Agency	Clinics, communal spaces	NGO staff, CHWs, traditonal birth attendants
Tanaka et al. (2008)	Mixed methods study	Tanzania	Refugees	Camp	Adolescents, Women >20 y, Pregnant women, Post-natal mothers, General population	Behavioural education, condom provision, HIV counselling and testing	NGO/UN Agency	Clinics, electronic/print, communal spaces	Peer educators, health workers,
Tayler-Smith et al. (2012)	Observational study	Liberia	Not displaced	N/A	WRA, general population	Psychological support, wound care, HIV/STI post-exposure prophylaxis, emergency contraception, hepatitis B and tetanus vaccinations, GBV behavioural education, referral for abortion services	NGO/UN Agency	Clinics, electronic/print, markets, schools	NGO staff
Tousaw et al. (2017)	Qualitative study	Thailand	Refugees	Camp	WRA	Referral for care, abortion and post-abortion care	NGO/UN Agency	NR	Counsellors
Tran et al. (2017)	Qualitative study	Burkina Faso, South Sudan	Refugees	Camp	Women >15 y,Pregnant women,Post-natal mothers	Training on GBV, emergency obstetric care (manual vacuum aspiration)	Healthcare system, NGO/UN Agency	NR	Health workers
Yiweza Tshipala et al. (2012)	Non-research	Côte d'Ivoire, Liberia, Ghana	IDPs, refugees	NR	General population	HIV prevention, treatment and follow-up care	NGO/UN Agency	Health posts	NR
Viswanathan et al. (2012)	Observational study	Afghanistan	Not displaced	N/A	Adolescents, Women (20-49 years), Pregnant	CHWs training	Healthcare system	Clinics	Health workers

Author	Report type	Country	Displacement status	Setting	Target population	Intervention	Delivery platform	Delivery site	Delivery personnel
					women, Post-natal mothers				
Von Roenne et al. (2010)	Non-research	Guinea	Refugees, hosts	Camp, Dispersed	WRA, Pregnant women, Post-natal mothers	Contraception provision, HIV/STI screening for referral, STI treatment and follow-up care, behavioural education	Healthcare system, NGO/UN Agency	Clinics,Hospitals, communal spaces, electronic/print	Nurses, SBAs, CHWs, other (drama groups)
Vu et al. (2017)	Mixed methods study	Kenya	Refugees	Camp	Women >15 y,Pregnant women,Post-natal mothers	GBV screening for referral, clinical care and counselling, behavioural education	NGO/UN Agency	Clinics	NGO staff, Health workers
Wayte et al. (2008)	Mixed methods study	East Timor	IDPs	Camp, Dispersed	WRA, Pregnant women	Contracpetion provision, training	Healthcare system, NGO/UN Agency	Hospitals, clinics, mobile clinics	Doctors, SBAs, obstetricians, health workers, NGO staff
West et al. (2016)	Qualitative study	Jordan	Refugees	Camp	WRA, Pregnant women, Post-natal mothers	Contraception provision	NGO/UN Agency	Clinics	Unreported
Wirtz et al. (2016)	Observational study	Colombia, Ethiopia	IDPs, refugees	Camp, Dispersed	Women >15 y,Pregnant women,Post-natal mothers	Screening for referral, GBV case management services, pschosocial support	NGO/UN Agency	Clinics, hospitals	Nurses, social workers, NGO staff
UNFPA (2018)	Non-research	Yemen	IDPs, Not displaced	NR	Adolescents	Psychosocial support for GBV	NGO/UN Agency	Unreported	NGO staff
UNFPA (2017)	Non-research	Iraq, Ukraine	IDPs, Hosts	Camp, Dispersed	Women >15 y, Pregnant women, Post-natal mothers	Dignity kits, Other GBV care (not specified)	NGO/UN Agency	Clinics, Mobile clinics	NGO staff
UNFPA (2016)	Non-research	Nigeria, Pakistan, Burma (Myanmar), Somalia	IDPs, Hosts	Camp, Dispersed	Adolescents, Women >20 y, Pregnant women, Post-natal mothers, General population	Family planning counselling and contraception provision, behavioural education, women friendly health spaces, training, HIV/STI Screening for referral with intent to treat, referral for care for obstetric fistulas	NGO/UN Agency	Clinics, Mobile Clinics, Health posts, home, electronic/print	Health workers, doctors, SBAs, CHWs, NGO/staff
Tanner S., O'Conner M. (2017)	Non-research	DRC, Ethiopia, Pakistan	IDPs, Refugees, Hosts	Camp, Dispersed	Adolescents	GBV Behavioural education (COMPASS programme), training	NGO/UN Agency	Communal spaces (safe space centres)	Health workers, female mentors, NGO staff

Author	Report type	Country	Displacement status	Setting	Target population	Intervention	Delivery platform	Delivery site	Delivery personnel
International Rescue Committee (IRC), (2016)	Non-research	DRC, Burma (Myanmar), Kenya	NR	N/A	Adolescents, Women (20-49 years), General population	Contraception provision, training, behavioural education	NGO/UN Agency	Clinics, Hospitals	Health workers/personnel
Yoshikawa, L. (2015)	Non-research	Jordan	Refugees, hosts	Camp, Dispersed	Children (5-9 years), Adolescents, Women >20 years, Pregnant women, Post-natal mothers, General population	GBV counselling, referral for care, cash transfer	NGO/UN Agency	Clinics (fixed Women's Centers), mobile clinics	NGO staff
Lilleston, P. et al (2016)	Non-research	Lebanon	IDPs, Refugees	Camp, Dispersed	Children (1-9 years), Adolescents, Women >20 years, Pregnant women ,Post-natal mothers	GBV Referral for care, behavioural education	NGO/UN Agency	Clinics, Hospitals, Place of worship, Schools, mobile clinics	Health workers, community members
International Rescue Committee (IRC), (2016)	Non-research	Chad	IDPs	Dispersed	Adolescents, Women >20 y, Pregnant women, Post-natal mothers, General population	Psychosocial support for GBV, behavioural education, distribution of dignity kits, cash transfers, training	NGO/UN Agency	Research centres, home	Health workers/personnel, NGO staff/researchers
Duroch, F.; Schulte-Hillen, C. (2014)	Non-research	DRC	IDPs	NR	General population	HIV/STI prevention, treatment and follow-up care, abortion and post-abortion care, emergency contraception	NGO/UN Agency	Clinics	NGO staff
Paik, K. (2014)	Non-research	Ethiopia, Tanzania, Uganda	Refugees	Camp	Adolescents, Pregnant women, Post-natal mothers	Safe spaces program, counselling for GBV	NGO/UN Agency	Communal spaces (Safe space centres in the community)	NGO staff
Women's Refugee Commission (2018)	Non-research	Jordan	Refugees	Camp, Dispersed	General population	Cash transfer, counselling for GBV (gender dialogue groups)	NGO/UN Agency	Health posts, home	NGO staff
Morren, G. et al (2016)	Non-research	Burundi	Not displaced	N/A	Women >15 y, Post-natal mothers	Obstetric fistula care (pre and post-operative care, surgery, physiotherapy, psychosocial support and education, nutritional support, follw-up care),	NGO/UN Agency	Hospitals, Home,Mobile clinics, Electronic/print, Communal spaces	Doctors, Nurses, NGO staff/researchers, fistula surgeons, physiotherapist, social workers, CHWs, skilled birth

Author	Report type	Country	Displacement status	Setting	Target population	Intervention	Delivery platform	Delivery site	Delivery personnel
						training, behavioural education, referral for care			attendants, other (journalists, caretakers)
UNHCR (2018)	Non-research	Rwanda	Refugees	Camp	Adolescents, Women >20 y, Pregnant women, Post-natal mothers, General population	Behavioural education on GBV	NGO/UN Agency	Mobile clinics	NGO staff, civic leaders, trained civilians
UNHCR (2015)	Non-research	Egypt, Jordan	Refugees	Camp, Dispersed	WRA,Pregnant women	Cash transfer, family planning counselling , behavioural education, referral for care	NGO/UN Agency	Clinics, Hospitals	Health workers, doctors, CHWs
Horn, R.; Seelinger, K.T. (2013)	Non-research	Kenya	IDPs, Refugees	Camp	Children (5-9 years), Adolescents, Women >20 years, Pregnant women, Post-natal mothers	Other GBV care (Temporary shelters)	NGO/UN Agency	Camps, transit centers, communal spaces (safe houses)	NGO staff
Feldman, S. et al (2013)	Non-research	Colombia	IDPs, Refugees	Camp	Women >15 y, Pregnant women, Post-natal mothers, General population	Psychosocial support for GBV, referral for care	NGO/UN Agency	NR	NGO staff, Trained civilians, Social workers
Freccero, J.; Seelinger, K.T. (2013)	Non-research	Thailand	IDPs, Refugees	Camp	Adolescents, Women >20 y, Pregnant women, Post-natal mothers	Counselling for GBV, referral for care, behavioural education	NGO/UN Agency	Clinics, Communal spaces (Safe House)	NGO staff/, Doctors, Nurses, Residential caretakers
UNICEF (2016)	Non-research	South Sudan	IDPs, Refugees, Not displaced	Camp, Dispersed	Adolescents, Women >20 y, Pregnant women, Post-natal mothers, General population	Training, behavioural education on GBV	NGO/UN Agency	Clinics	NGO staff
UNICEF (2016)	Non-research	Pakistan	IDPs, Refugees	Camp	Children (1-9 years), Adolescents, Women >20 years, Pregnant women, Post-natal mothers	Behavioural education on GBV	NGO/UN Agency	Communal spaces (private spaces)	Trained civilians

BMJ Publishing Group Limited (BMJ) disclaims all liability and responsibility arising from any reliance placed on this supplemental material which has been supplied by the author(s)

Author	Report type	Country	Displacement status	Setting	Target population	Intervention	Delivery platform	Delivery site	Delivery personnel
UNICEF (2016)	Non-research	Lebanon	Refugees, hosts	NR	Infants (0-12 mo), Children (1-9 y), Adolescents, Women >20 y, Pregnant women, Post-natal mothers	Standard integrated package of GBViE services : counselling, referral for care, behavioural education, distribution of dignity kits, safe shelters, training	NGO/UN Agency	Clinics, Hospitals, Communal spaces, Camps, Electronic/print, Health posts, Mobile clinics, Place of worship, Schools	NGO staff, other (Health, legal, social, and justice actors)
UNICEF (2013)	Non-research	Pakistan	IDPs, Hosts	Camp, Dispersed	General population	Training on GBV	NGO/UN Agency	Clinics	NGO staff
UNICEF (2016)	Non-research	Somalia	IDPs, Not displaced	Camp, Dispersed	Adolescents, Women >20 y, Pregnant women, Post-natal mothers, General population	minimum package of essential services GBV care (medical care, psychosocial support, safety and legal assistance, distribution of dignity kits, safe shelters), training, behavioural education (communities care programme)	NGO/UN Agency	Clinics, communal spaces, research centres	NGO staff, Trained civilians, civic leaders
UNICEF (2016)	Non-research	Central African Republic	IDPs, Not displaced	Camp, Dispersed	Children (1-9 years), Adolescents, Women >20 years, Pregnant women, Post-natal mothers, General population	GBV care (psychosocial support, referral for care, distribution of dignity kits, behavioural education, legal support), training	NGO/UN Agency	Home, communal spaces (listening centres), electronic/print, hospitals, research centres	NGO staff, civic leaders,civilians
UNICEF (2016)	Non-research	Central African Republic	IDPs	Camp, Dispersed	Pregnant women	HIV Counselling	NGO/UN Agency	NR	NGO staff
UNICEF (2013)	Non-research	DRC	IDPs	Camp, Dispersed	General population	GBV screening for referral	NGO/UN Agency	Health posts	NGO staff

NR: Not reported

Munyuzangabo M, *et al. BMJ Global Health* 2020; 5:e002206. doi: 10.1136/bmjgh-2019-002206

Appendix C: Reported coverage and effectiveness of SRH interventions

Author & year	Country of study	Age group	Intervention	Report type	Outcome	Description of outcome	Effect measure	Sample size	Post-intervention numbers (95% CI)	Displacement status	Settlement settings	Delivery personnel	Site of delivery
**Family planning services**													
Chukwumalu et al. 2017	Somalia	Women 15-49 years	Abortion and post-abortion care	Observational study	Coverage	Proportion of women treated with MVA out of those who received PAC services at the four Save the Children supported health facilities between 2013 - 2015	%	1111	57.0 (54.1, 59.9)	IDPs	NR	Nurses, SBAs, health workers	Clinics, hospitals
Chukwumalu et al. 2017	Somalia	Women 15-49 years	Abortion and post-abortion care	Observational study	Coverage	Proportion of women treated with Misoprostol out of those who received PAC services at the four Save the Children supported health facilities between 2013 - 2015	%	1111	24.0 (21.5, 26.5)	IDPs	NR	Nurses, SBAs, health workers	Clinics, hospitals
Kinaro et al. 2009	Sudan	Women 15-49 years	Abortion and post-abortion care	Mixed methods	Coverage	Proportion of surveyed women who received post-abortion care out of women admitted at the study hospitals for abortion or post-abortion care between October 2007 and January 2008	%	726	96.7 (95.4, 98.0)	IDPs, not displaced	Dispersed	Doctors	Hospitals
Tousaw et al. 2017	Thailand	Women 15-49 years	Abortion and post-abortion care	Qualitative study	Coverage	Proportion of women who were referred for care and received safe and legal abortions in either a Thai public hospital or a Thai private clinic, after accessing The Safe Abortion Referral Programme (SARP)	%	81	64.0 (53.5, 74.5)	Refugees, migrants	Camp	NR	Clinics, hospitals
Adam et al. 2016	Sudan	Women 15-49 years	Contraception provision	Observational study	Effectiveness	Adjusted odds ratio of current use of any modern family planning method in women who had experienced pregnancy during the last 2 years, comparing endline to baseline survey	OR	640	2.8 (2.0, 4.1)	IDPs	Camp	NR	Clinics
Casey et al. 2013	Uganda	Women 15-49 years	Contraception provision	Observational study	Effectiveness	Adjusted odds ratio of current use of any modern family planning method in women aged 15-49 years surveyed at endline (2010) compared to those surveyed at baseline (2007)	OR	1768	3.34 (2.27, 4.92)	IDPs	Both settings	Doctors, nurses, SBAs	Clinics, mobile clinics
Casey et al. 2013	Uganda	Women 15-49 years	Contraception provision	Observational study	Effectiveness	Adjusted odds ratio of current use of a long-acting and permanent FP methods (LAPM) in women aged 15-49 years surveyed at endline (2010) compared to those surveyed at baseline (2007)	OR	1768	9.45 (3.98, 22.42)	IDPs	Both settings	Doctors, nurses, SBAs	Clinics, mobile clinics
Casey et al. 2013	Uganda	Women 15-49 years	Contraception provision	Observational study	Effectiveness	Adjusted odds ratio in unmet need for FP in women aged 15-49 years surveyed at endline (2010) compared to those surveyed at baseline (2007)	OR	1768	0.47 (0.37,0.6)	IDPs	Both settings	Doctors, nurses, SBAs	Clinics, mobile clinics

Munyuzangabo M, *et al. BMJ Global Health* 2020; 5:e002206. doi: 10.1136/bmjgh-2019-002206

Author & year	Country of study	Age group	Intervention	Report type	Outcome	Description of outcome	Effect measure	Sample size	Post-intervention numbers (95% CI)	Displacement status	Settlement settings	Delivery personnel	Site of delivery
Casey et al. 2017	DRC	Women 15-49 years	Contraception provision	Observational study	Effectiveness	Adjusted odds ratio of current use of any modern method of contraception in women aged 15-49 years surveyed at baseline (2008) compared to those surveyed at endline (2010)	OR	1171	2.03 (1.3, 3.2)	Not displaced	N/A	Health workers	Clinics, hospitals
Casey et al. 2017	DRC	Women 15-49 years	Contraception provision	Observational study	Coverage	Reported current use of any modern method of contraception in women aged 15-49 years surveyed at baseline (2008)	%	607	3.1 (2, 4.2)	Not displaced	N/A	Health workers	Clinics, hospitals
Casey et al. 2017	DRC	Women 15-49 years	Contraception provision	Observational study	Coverage	Reported current use of any modern method of contraception in women aged 15-49 years surveyed at endline (2010)	%	564	5.9 (4.5, 7.4)	Not displaced	N/A	Health workers	Clinics, hospitals
Chukwumalu et al. 2017	Somalia	Women 15-49 years	Contraception provision	Observational study	Coverage	Proportion of women who opted to use oral contraceptive pills out of those who received PAC services at the four Save the Children supported health facilities between 2013 - 2015	%	1111	34.0 (31.21, 36.79)	IDPs	NR	Nurses, SBAs, health workers	Clinics, hospitals
Chukwumalu et al. 2017	Somalia	Women 15-49 years	Contraception provision	Observational study	Coverage	Proportion of women who opted to use injectables out of those who received PAC services at the four Save the Children supported health facilities between 2013 - 2015	%	1111	27.0 (24.39, 29.61)	IDPs	NR	Nurses, SBAs, health workers	Clinics, hospitals
Chukwumalu et al. 2017	Somalia	Women 15-49 years	Contraception provision	Observational study	Coverage	Proportion of women who opted to use an IUD out of those who received PAC services at the four Save the Children supported health facilities between 2013 - 2015	%	1111	14.0 (11.96, 16.04)	IDPs	NR	Nurses, SBAs, health workers	Clinics, hospitals
Chukwumalu et al. 2017	Somalia	Women 15-49 years	Contraception provision	Observational study	Coverage	Proportion of women who opted to use an implant out of those who received PAC services at the four Save the Children supported health facilities between 2013 - 2015	%	1111	11.0 (9.16, 12.84)	IDPs	NR	Nurses, SBAs, health workers	Clinics, hospitals
Curry et al. 2015	Chad	Women 15-49 years	Contraception provision	Non-research	Coverage	Number of women using implants among the new modern contraceptive users found through the SAFPAC- supported facilities between July 2011 and December 2013	Number	21191	13201	IDPs, refugees	NR, Camp	CHWs, doctors, nurses	Clinics, hospitals
Curry et al. 2015	Chad	Women 15-49 years	Contraception provision	Non-research	Coverage	Number of women using IUDs among the new modern contraceptive users found through the SAFPAC- supported facilities between July 2011 and December 2013	Number	21191	2189	IDPs, refugees	NR, Camp	CHWs, doctors, nurses	Clinics, hospitals
Curry et al. 2015	Chad	Women 15-49 years	Contraception provision	Non-research	Coverage	Number of women using other modern methods among the new modern contraceptive users found through the	Number	21191	5841	IDPs, refugees	NR, Camp	CHWs, doctors, nurses	Clinics, hospitals

Author & year	Country of study	Age group	Intervention	Report type	Outcome	Description of outcome	Effect measure	Sample size	Post-intervention numbers (95% CI)	Displacement status	Settlement settings	Delivery personnel	Site of delivery
						SAFPAC- supported facilities between July 2011 and December 2013							
Curry et al. 2015	Chad	Women 15-49 years	Contraception provision	Non-research	Coverage	Proportion of women using LARCs among all women who are new modern contraceptive users found through the SAFPAC- supported facilities between July 2011 and December 2013	%	21191	72.0 (71.40, 72.60)	IDPs, refugees	NR, Camp	CHWs, doctors, nurses	Clinics, hospitals
Curry et al. 2015	DRC	Women 15-49 years	Contraception provision	Non-research	Coverage	Number of women using implants among the new modern contraceptive users found through the SAFPAC- supported facilities between July 2011 and December 2013	Number	14869	9132	IDPs, refugees	NR, Camp	CHWs, doctors, nurses	Clinics, hospitals
Curry et al. 2015	DRC	Women 15-49 years	Contraception provision	Non-research	Coverage	Number of women using IUDs among the new modern contraceptive users found through the SAFPAC- supported facilities between July 2011 and December 2013	Number	14869	2289	IDPs, refugees	NR, Camp	CHWs, doctors, nurses	Clinics, hospitals
Curry et al. 2015	DRC	Women 15-49 years	Contraception provision	Non-research	Coverage	Number of women using other modern methods among the new modern contraceptive users found through the SAFPAC- supported facilities between July 2011 and December 2013	Number	14869	3310	IDPs, refugees	NR, Camp	CHWs, doctors, nurses	Clinics, hospitals
Curry et al. 2015	DRC	Women 15-49 years	Contraception provision	Non-research	Coverage	Proportion of women using LARCs among all women who are new modern contraceptive users found through the SAFPAC- supported facilities between July 2011 and December 2013	%	14869	78.0 (77.33, 78.67)	IDPs, refugees	NR, Camp	CHWs, doctors, nurses	Clinics, hospitals
Curry et al. 2015	Djibouti	Women 15-49 years	Contraception provision	Non-research	Coverage	Number of women using implants among the new modern contraceptive users found through the SAFPAC- supported facilities between July 2011 and December 2013	Number	575	6	Refugees	Camp	CHWs, doctors, nurses	Clinics, hospitals
Curry et al. 2015	Djibouti	Women 15-49 years	Contraception provision	Non-research	Coverage	Number of women using IUDs among the new modern contraceptive users found through the SAFPAC- supported facilities between July 2011 and December 2013	Number	575	0	Refugees	Camp	CHWs, doctors, nurses	Clinics, hospitals
Curry et al. 2015	Djibouti	Women 15-49 years	Contraception provision	Non-research	Coverage	Number of women using other modern methods among the new modern contraceptive users found through the SAFPAC- supported facilities between July 2011 and December 2013	Number	575	569	Refugees	Camp	CHWs, doctors, nurses	Clinics, hospitals
Curry et al. 2015	Djibouti	Women 15-49 years	Contraception provision	Non-research	Coverage	Proportion of women using LARCs among all women who are new modern contraceptive users found through the	%	575	1.0 (0.19, 1.81)	Refugees	Camp	CHWs, doctors, nurses	Clinics, hospitals

Author & year	Country of study	Age group	Intervention	Report type	Outcome	Description of outcome	Effect measure	Sample size	Post-intervention numbers (95% CI)	Displacement status	Settlement settings	Delivery personnel	Site of delivery
						SAFPAC- supported facilities between July 2011 and December 2013							
Curry et al. 2015	Mali	Women 15-49 years	Contraception provision	Non-research	Coverage	Number of women using implants among the new modern contraceptive users found through the SAFPAC- supported facilities between July 2011 and December 2013	Number	3093	1317	IDPs	NR	CHWs, doctors, nurses	Clinics, hospitals
Curry et al. 2015	Mali	Women 15-49 years	Contraception provision	Non-research	Coverage	Number of women using IUDs among the new modern contraceptive users found through the SAFPAC- supported facilities between July 2011 and December 2013	Number	3093	270	IDPs	NR	CHWs, doctors, nurses	Clinics, hospitals
Curry et al. 2015	Mali	Women 15-49 years	Contraception provision	Non-research	Coverage	Number of women using other modern methods among the new modern contraceptive users found through the SAFPAC- supported facilities between July 2011 and December 2013	Number	3093	1506	IDPs	NR	CHWs, doctors, nurses	Clinics, hospitals
Curry et al. 2015	Mali	Women 15-49 years	Contraception provision	Non-research	Coverage	Proportion of women using LARCs among all women who are new modern contraceptive users found through the SAFPAC- supported facilities between July 2011 and December 2013	%	3093	51.0 (49.24, 52.76)	IDPs	NR	CHWs, doctors, nurses	Clinics, hospitals
Curry et al. 2015	Pakistan	Women 15-49 years	Contraception provision	Non-research	Coverage	Number of women using implants among the new modern contraceptive users found through the SAFPAC- supported facilities between July 2011 and December 2013	Number	12888	298	IDPs	NR	CHWs, doctors, nurses	Clinics, hospitals
Curry et al. 2015	Pakistan	Women 15-49 years	Contraception provision	Non-research	Coverage	Number of women using IUDs among the new modern contraceptive users found through the SAFPAC- supported facilities between July 2011 and December 2013	Number	12888	3432	IDPs	NR	CHWs, doctors, nurses	Clinics, hospitals
Curry et al. 2015	Pakistan	Women 15-49 years	Contraception provision	Non-research	Coverage	Number of women using other modern methods among the new modern contraceptive users found through the SAFPAC- supported facilities between July 2011 and December 2013	Number	12888	9158	IDPs	NR	CHWs, doctors, nurses	Clinics, hospitals
Curry et al. 2015	Pakistan	Women 15-49 years	Contraception provision	Non-research	Coverage	Proportion of women using LARCs among all women who are new modern contraceptive users found through the SAFPAC- supported facilities between July 2011 and December 2013	%	12888	29.0 (28.22, 29.78)	IDPs	NR	CHWs, doctors, nurses	Clinics, hospitals
Huber at al. 2010	Afghanistan	Women 15-49 years	Contraception provision	Observational study	Coverage	Number of women who were contraceptive users before the implementation of the ACU project in all women who were surveyed all 3 sites	Number	3708	532	Not displaced	N/A	CHWs	NR

Author & year	Country of study	Age group	Intervention	Report type	Outcome	Description of outcome	Effect measure	Sample size	Post-intervention numbers (95% CI)	Displacement status	Settlement settings	Delivery personnel	Site of delivery
Huber at al. 2010	Afghanistan	Women 15-49 years	Contraception provision	Observational study	Coverage	Number of women who were contraceptive users after the implementation of the ACU project in all women who were sureveyed all 3 sites	Number	3708	1469	Not displaced	N/A	CHWs	NR
Kinaro et al. 2009	Sudan	Women 15-49 years	Contraception provision	Mixed methods	Coverage	Proportion of surveyed women who went home with a contraceptive method out of women admitted at the study hospitals for abortion or post-abortion care between October 2007 and January 2008	%	726	12.3 (9.91, 14.69)	IDPs, not displaced	Dispersed	Doctors	Hospitals
McGinn et al. 2011	DRC	Women 15-49 years	Contraception provision	Observational study	Coverage	Contraceptive prevalence rate (modern methods) among married or in-union WRA attending member clinics of the RAISE initiative in Eastern Congo	Rate	558	3.2	NR	NR	NR	Clinics, hospitals
McGinn et al. 2011	Sudan	Women 15-49 years	Contraception provision	Observational study	Coverage	Contraceptive prevalence rate (modern methods) among married or in-union WRA attending member clinics of the RAISE initiative in North Darfur	Rate	738	2.3	IDPs, not displaced	Both settings, N/A	NR	Clinics, hospitals
McGinn et al. 2011	Sudan	Women 15-49 years	Contraception provision	Observational study	Coverage	Contraceptive prevalence rate (modern methods) among married or in-union WRA attending member clinics of the RAISE initiative in West Darfur	Rate	559	12	IDPs, not displaced	Both settings, N/A	NR	Clinics, hospitals
McGinn et al. 2011	Sudan	Women 15-49 years	Contraception provision	Observational study	Coverage	Contraceptive prevalence rate (modern methods) among married or in-union WRA attending member clinics of the RAISE initiative in South Darfur	Rate	690	1.7	IDPs, not displaced	Both settings, N/A	NR	Clinics, hospitals
McGinn et al. 2011	Sudan	Women 15-49 years	Contraception provision	Observational study	Coverage	Contraceptive prevalence rate (modern methods) in among married or in-union WRA attending member clinics of the RAISE initiative Southern Sudan	Rate	420	1.9	IDPs, not displaced	Both settings, N/A	NR	Clinics, hospitals
McGinn et al. 2011	Uganda	Women 15-49 years	Contraception provision	Observational study	Coverage	Contraceptive prevalence rate (modern methods) among married or in-union WRA attending member clinics of the RAISE initiative in Northern Uganda	Rate	1238	16.2	NR	NR	NR	Clinics
Nattabi et al. 2011	Uganda	Women 15-49 years	Contraception provision	Mixed methods	Coverage	Proportion of women who reported currently using any form of family planning out of the surveyed women living with HIV/AIDS attending HIV clinics	%	238	25 (19.50, 30.50)	IDPs, Not displaced	Both settings	Health workers	Clinics, Mobile clinics
von Roenne et al. 2010	Guinea	Women 15-49 years	Contraception provision	Non-research	Coverage	Couple years protection (CYPs) achieved by the distribution of contraceptives by RHG nurses in 1999	CYP	37456	5315	Refugees, hosts	Both settings	Nurses, SBAs, NGO staff	Clinics, hospitals
Adam et al. 2016	Sudan	Women 15-49 years	Counselling	Observational study	Coverage	Women receiving home-based FP counseling among those who gave birth in the last 2 years	%	640	59.8 (56.0, 63.60)	IDPs	Camp	CHWs	Home

Author & year	Country of study	Age group	Intervention	Report type	Outcome	Description of outcome	Effect measure	Sample size	Post-intervention numbers (95% CI)	Displacement status	Settlement settings	Delivery personnel	Site of delivery
Chukwumalu et al. 2017	Somalia	Women 15-49 years	Counselling	Observational study	Coverage	Proportion of women counselled for post abortion contraception out of those who received PAC services at the four Save the Children supported health facilities between 2013 - 2015	%	1111	98 (97.17,98.82)	IDPs	NR	Nurses, SBAs, health workers	Clinics, hospitals
Kinaro et al. 2009	Sudan	Women 15-49 years	Counselling	Mixed methods	Coverage	Proportion of surveyed women who received contraceptive counselling out of women admitted at the study hospitals for abortion or post-abortion care between October 2007 and January 2008	%	726	35.3 (31.82, 38.78)	IDPs, not displaced	Dispersed	Doctors	Hospitals
Nattabi et al. 2011	Uganda	Women 15-49 years	Counselling	Mixed methods	Coverage	Proportion of women who reported ever discussing family planning with health workers out of the surveyed women living with HIV/AIDS attending HIV clinics	%	281	59 (53.25, 64.75)	IDPs, Not displaced	Both settings	Health workers	Clinics, Mobile clinics
Tousaw et al. 2017	Thailand	Women 15-49 years	Screening for referral/referral for care	Qualitative study	Coverage	Number of women from Burmese communities who accessed the Safe Abortion Referral Programme (SARP)	Number	0	81	Refugess, migrants	Camp	Counsellors	NR
Viswanathan et al. 2012	Afghanistan	Women 10-49 years	Training	Observational study	Effectiveness	Odds of using a modern method of contraception in women in villages who had at least one female CHW compared to those that didn't have any CHW, among all surveyed women aged 10-49 years who were married and non-pregnant	OR	6269	1.61 (1.21, 2.15)	Not displaced	N/A	Health workers	Clinics
**Gender-based violence**													
Tayler-Smith et al. 2012	Liberia	Women 15-49 years	Abortion and post-abortion care	Observational study	Coverage	Proportion of sexual violence survivors who received an abortion due to a pregnancy caused by sexual trauma out of those who requested one.	%	48	81.0 (69.91, 92.10)	Not Displaced	N/A	NGO staff	Clinics
Tayler-Smith et al. 2012	Liberia	Women 10-49 years	Contraception provision	Observational study	Coverage	Proportion of sexual violence survivors aged 10 or older who were given emergency contraception out of those who presented at the SV clinics within 120h of their aggression	%	425	49.0 (44.25, 53.75)	Not Displaced	N/A	NGO staff	Clinics
Loko Roka et al. 2014	DRC	Women 12-45 years	Contraception provision	Observational study	Coverage	Proportion of sexual violence survivors aged 12-45 years who started emergency contraception in Masisi out of those who were eligible (presenting within 5 days)	%	274	91.0 (87.61, 94.39)	NR	N/A	Doctors, nurses	Clinics, Hospitals
Loko Roka et al. 2014	DRC	Women 12-45 years	Contraception provision	Observational study	Coverage	Proportion of of sexual violence survivors aged 12-45 years who started emergency contraception in Niangara out of those	%	53	87.0 (77.95, 96.05)	NR	N/A	Doctors, nurses	Clinics, Hospitals

Author & year	Country of study	Age group	Intervention	Report type	Outcome	Description of outcome	Effect measure	Sample size	Post-intervention numbers (95% CI)	Displacement status	Settlement settings	Delivery personnel	Site of delivery
						who were eligible (presenting within 5 days)							
Gupta et al. 2013	Cote d'Ivoire	Women 15-65+ years	Counselling	Observational study	Effectiveness	Odds of reporting physical and/or sexual IPV in the past year in women who received VSLA + GDG compared to women who only received the VSLA intervention (ITT analysis)	OR	1788	0.92 (0.58, 1.47)	NR	NR	NGO staff	NR
Gupta et al. 2013	Cote d'Ivoire	Women 15-65+ years	Counselling	Observational study	Effectiveness	Adjusted odds of reporting physical and/or sexual IPV in the past year in women who received VSLA + GDG with high adherence compared to women who only received the VSLA intervention (PP analysis)	OR	1769	0.64 (0.35, 1.16)	NR	NR	NGO staff	NR
Gupta et al. 2013	Cote d'Ivoire	Women 15-65+ years	Counselling	Observational study	Effectiveness	Adjusted odds of reporting physical IPV in the past year in women who received VSLA + GDG with high adherence compared to women who only received the VSLA intervention (PP analysis)	OR	1769	0.45 (0.21, 0.94)	NR	NR	NGO staff	NR
Tayler-Smith et al. 2012	Liberia	Children 0-14 years, women 15-65+ years	HIV prevention, treatment and follow-up care	Observational study	Coverage	Proportion of sexual violence survivors that presented within 72 h of the attack at SV clinics and received post-exposure prophylaxis for HIV	%	619	78.0 (74.74, 81.26)	Not Displaced	N/A	NGO staff	Clinics
Tayler-Smith et al. 2012	Liberia	Children 0-14 years, women 15-65+ years	HIV prevention, treatment and follow-up care	Observational study	Coverage	Proportion of sexual violence survivors that presented within 72 h of the attack at SV clinics and received and completed the post-exposure prophylaxis for HIV	%	482	58.0 (53.60, 62.41)	Not Displaced	N/A	NGO staff	Clinics
O'Callaghan et al. 2013	DRC	Adolescents 12-17 years	Mental health intervention	Randomized controlled tiral	Effectiveness	Effect size in reduction of trauma symptoms among adolescent girls aged 12-17 years who received trauma-focused cognitive behavioral therapy compared to the wait-list control group	F value	24	52.708	IDPs, Not displaced	NR, N/A	Health workers	Schools
Bass et al. 2013	DRC	Women 10-65+ years, pregnant, postnatal mothers	Mental health intervention	Quasi-experimental/non-RCT	Effectiveness	Mean scores of depression and anxiety at baseline in women who received the individual support group out of all surveyed women who had experienced or witnessed sexual violence and had high levels of PTSD symptoms and combined depression and anxiety symptoms	Mean	248	2.2	NR	NR	Psychosocial assistants	Research centre

Author & year	Country of study	Age group	Intervention	Report type	Outcome	Description of outcome	Effect measure	Sample size	Post-intervention numbers (95% CI)	Displacement status	Settlement settings	Delivery personnel	Site of delivery
Bass et al. 2013	DRC	Women 10-65+ years, pregnant, postnatal mothers	Mental health intervention	Quasi-experimental/non-RCT	Effectiveness	Mean scores of depression and anxiety at the end of treatment in women who received the individual support group out of all surveyed women who had experienced or witnessed sexual violence and had high levels of PTSD symptoms and combined depression and anxiety symptoms	Mean	248	1.7	NR	NR	Psychosocial assistants	Research centre
Bass et al. 2013	DRC	Women 10-65+ years, pregnant, postnatal mothers	Mental health intervention	Quasi-experimental/non-RCT	Effectiveness	Mean scores of depression and anxiety at baseline in women who received cognitive processing therapy out of all surveyed women who had experienced or witnessed sexual violence and had high levels of PTSD symptoms and combined depression and anxiety symptoms	Mean	157	2	NR	NR	Psychosocial assistants	Research centre
Bass et al. 2013	DRC	Women 10-65+ years, pregnant, postnatal mothers	Mental health intervention	Quasi-experimental/non-RCT	Effectiveness	Mean scores of depression and anxiety at the end of treatment in women who received cognitive processing therapy out of all surveyed women who had experienced or witnessed sexual violence and had high levels of PTSD symptoms and combined depression and anxiety symptoms	Mean	157	0.8	NR	NR	Psychosocial assistants	Research centre
Mankuta et al. 2012	DRC	Women 10-65+, pregnant, postnatal mothers	Mental health intervention	Observational study	Coverage	Number of women who were offered psychological interventions out of the ones who had attended the clinics and been diagnosed with severe PTSD	Number	52	23	NR	NR	Doctors, nurses, OB/GYN, mental health professional	Health posts
Loko Roka et al. 2014	DRC	Women 12-45 years	Screening for referral/referral for care	Observational study	Coverage	Proportion of sexual violence survivors aged 12-45 years who received a Pregnancy test in Masisi out of those that presented for care	%	424	96.0 (94.13, 97.87)	NR	N/A	Doctors, nurses	Clinics, Hospitals
Loko Roka et al. 2014	DRC	Women 12-45 years	Screening for referral/referral for care	Observational study	Coverage	Proportion of sexual violence survivors aged 12-45 years who received a Pregnancy test in Niangara out of those that presented for care	%	148	88.0 (82.76, 93.24)	NR	N/A	Doctors, nurses	Clinics, Hospitals

BMJ Publishing Group Limited (BMJ) disclaims all liability and responsibility arising from any reliance placed on this supplemental material which has been supplied by the author(s)

Author & year	Country of study	Age group	Intervention	Report type	Outcome	Description of outcome	Effect measure	Sample size	Post-intervention numbers (95% CI)	Displacement status	Settlement settings	Delivery personnel	Site of delivery
Vu et al. 2017	Kenya	Women 15-65+ years	Screening for referral/referral for care	Mixed methods	Coverage	Proportion of potentially eligible participants who were screened for GBV using the ASIST-GBV tool out of women who presented at the IRC clinics	%	64212	15.0 (14.74, 15.28)	Refugees	Camp	NGO staff, health workers	Clinics
Vu et al. 2017	Kenya	Women 15-65+ years	Screening for referral/referral for care	Mixed methods	Coverage	Number women who attended referral services at the IRC support centre out of the women who tested positive for GBV using the ASIST GBV tool	Number	0	234	Refugees	Camp	NGO staff, health workers	Clinics
Wirtz et al. 2016	Ethiopia	Women 15-65+ years	Screening for referral/referral for care	Observational study	Coverage	Proportion of refugee women who accepted referral for GBV services at the time it was offered out of all women who presented at the clinics and screened positive for GBV using the ASIST GBV tool in the last 12 months	%	244	43.8 (37.57, 50.03)	Refugees	Both settings	NGO staff, health workers	Clinics
Wirtz et al. 2016	Colombia	Women 18-65+ years	Screening for referral/referral for care	Observational study	Coverage	Proportion of IDP women who accepted referral for GBV services at the time it was offered out of all women who presented at the clinics and screened positive for GBV using the ASIST GBV tool in the last 12 months	%	319	74.2 (69.40, 79.0)	IDPs	Dispersed	Nurses	Clinics, hospitals
Loko Roka et al. 2014	DRC	Women 12-65+ years	Vaccinations	Observational study	Coverage	Proportion of sexual violence survivors who received tetanus vaccination (TT1) out of those that presented for care in Masisi	%	491	68.0 (63.87, 72.13)	NR	N/A	Doctors, nurses	Clinics, Hospitals
Loko Roka et al. 2014	DRC	Women 12-65+ years	Vaccinations	Observational study	Coverage	Proportion of sexual violence survivors who received tetanus vaccination (TT2) out of those who had received the first dose (TT1) in Masisi	%	334	18.0 (13.88, 22.12)	NR	N/A	Doctors, nurses	Clinics, Hospitals
Loko Roka et al. 2014	DRC	Women 12-65+ years	Vaccinations	Observational study	Coverage	Proportion of sexual violence survivors who received tetanus vaccination (TT1) out of those that presented for care in Niangara	%	180	77.0 (70.85, 83.15)	NR	N/A	Doctors, nurses	Clinics, Hospitals
Loko Roka et al. 2014	DRC	Women 12-65+ years	Vaccinations	Observational study	Coverage	Proportion of sexual violence survivors who received tetanus vaccination (TT2) out of those who had received the first dose (TT1) in Niangara	%	138	26.0 (18.68, 33.32)	NR	N/A	Doctors, nurses	Clinics, Hospitals
**General reproductive health**													
von Roenne et al. 2010	Guinea	Women 15-65+ years	Counselling	Non-research	Coverage	Proportion of community members who received SRH counselling from the RHG facilitators out of all the individual contacts they had in 1999	%	87260	94.0 (93.84, 94.16)	Refugees, hosts	Both settings	NGO staff	Camps, communal spaces

Author & year	Country of study	Age group	Intervention	Report type	Outcome	Description of outcome	Effect measure	Sample size	Post-intervention numbers (95% CI)	Displacement status	Settlement settings	Delivery personnel	Site of delivery
Morren et al. 2016	Burundi	Women 15-65+ years, postnatal mothers	Fistula surgery	Non-research	Effectiveness	Proportion of women with fistulas that were able to be closed and who were continent after their first surgery out of the women who presented at the Gitega Fistula Centre	%	1020	61.0 (58.01, 64.0)	Not displaced	N/A	Doctors, nurses, surgeons	Hospitals
Morren et al. 2016	Burundi	Women 15-65+ years, postnatal mothers	Fistula surgery	Non-research	Effectiveness	Proportion of women with fistulas that were closed and who were continent out of the women who presented at the Gitega Fistula Centre and needed a repeated surgery	%	468	41.0 (36.54, 45.46)	Not displaced	N/A	Doctors, nurses, surgeons	Hospitals
Shaikh et al. 2008	Somalia	Women 15-49 years, pregnant women, postnatal mothers	Screening for referral/referral for care	Non-research	Coverage	Number of consultations provided to women and children during a month long project which provided free healthcare services through a mobile clinic in IDP camps	Number	0	3095	IDPs	Camp	Nurses, SBAs	Mobile clinics
McGinn et al. 2006	Guinea	Women 15-65+ years, pregnant, postnatal mothers	Training	Observational study	Coverage	Number of refugee women who underwent Reproductive Health Literacy (RHL) training between 1999 to 2001	Number	0	2325	Refugees	Camp	NGO staff, researchers	NR
**HIV/STIs**													
Malemo Kalisya et al. 201	DRC	Children 0-18 years	HIV prevention, treatment and follow-up care	Observational study	Coverage	Proportion of sexual assault survivors aged 18 years or younger who received post-exposure prophylaxis out of those who presented within 72hours of assault at the HEAL Africa Hospital between 2006 and 2008	%	193	67.0 (60.37, 73.63)	NR	NR	NR	Hospitals
Kim et al. 2009	DRC	Women 15-49 years	Distribution of referral vouchers	Observational study	Coverage	Proportion of women from the river communities who took up VCT out of the surveyed women who had received referral vouchers for free VCT services	%	1059	20.0 (17.59, 22.41)	IDPs, not displaced, returning refugees	Both settings	NR	Clinics

Author & year	Country of study	Age group	Intervention	Report type	Outcome	Description of outcome	Effect measure	Sample size	Post-intervention numbers (95% CI)	Displacement status	Settlement settings	Delivery personnel	Site of delivery
Kim et al. 2009	DRC	Women 15-49 years	Distribution of referral vouchers	Observational study	Coverage	Proportion of women from the IDP camp who took up VCT out of the surveyed women who had received referral vouchers for free VCT services	%	225	90.0 (86.08, 93.92)	IDPs, not displaced, returning refugees	Both settings	NR	Clinics
Bannink-Mbazzi et al. 2013	Uganda	Children (6 weeks - 18 months)	STI screening for referral	Observational study	Coverage	Number of children born to HIV-infected mothers who had been tested for HIV between the ages of 6 weeks and 18 months at the MOH/AVSI health facilities between 2004 and 2011	Number	0	4436	IDPs, not displaced	Camp	NR	Clinics
Malemo Kalisya et al. 201	DRC	Children 0-18 years	STI screening for referral	Observational study	Coverage	Proportion of sexual violence survivors aged 18 or younger screened for HIV out of those who presented at the HEAL Africa Hospital between 2006 and 2008	%	440	70.0 (65.72, 74.28)	NR	NR	NR	Hospitals
