# Supplementary material for: Delivery of sexual and reproductive health interventions in conflict settings: a systematic review
Source: BMJ Glob Health. 2020 Jul 21;5(Suppl 1):e002206. doi: 10.1136/bmjgh-2019-002206 (PMC7375437; doi:10.1136/bmjgh-2019-002206)
Supplement: Supplementary data [file bmjgh-2019-002206supp002.pdf]

Publications included in the review

Adam IF. Evidence from cluster surveys on the association between home-based counseling and use of family planning in conflict-affected Darfur. Int J Gynaecol Obstet. 2016;133(2):221-5.

Adam IF, Nakamura K, Kizuki M, Al Rifai R, Vanching U. Relationship between implementing interpersonal communication and mass education campaigns in emergency settings and use of reproductive healthcare services: Evidence from Darfur, Sudan. BMJ open. 2015;5(9):e008285.

Anonymous. GPA joins emergency efforts in Rwanda. Global AIDSnews : the newsletter of the World Health Organization Global Programme on AIDS. 1994(4):1-3.

Balsara ZP, Wu I, Marsh DR, Ihsan AT, Nazir R, Owoso E, et al. Reproductive tract disorders among Afghan refugee women attending health clinics in Haripur, Pakistan. Journal of health, population, and nutrition. 2010;28(5):501-8.

Bannink-Mbazzi F, Lowicki-Zucca M, Ojom L, Kabasomi SV, Esiru G, Homsy J. High PMTCT program uptake and coverage of mothers, their partners, and babies in northern Uganda: achievements and lessons learned over 10 years of implementation (2002-2011). J Acquir Immune DeficSyndr. 2013;62(5):e138-45.

Bass JK, Annan J, McIvor Murray S, Kaysen D, Griffiths S, Cetinoglu T, et al. Controlled trial of psychotherapy for Congolese survivors of sexual violence. N Engl J Med. 2013;368(23):2182-91.

Benage M, Greenough PG, Vinck P, Omeira N, Pham P. An assessment of antenatal care among Syrian refugees in Lebanon. Conflict and health. 2015;9:8.

Benjamin JA. AIDS prevention for refugees. The case of Rwandans in Tanzania. Aidscaptions. 1996;3(2):4-9.

Bhardwaj A, Bourey C, Rai S, Adhikari RP, Worthman CM, Kohrt BA. Interpersonal violence and suicidality among former child soldiers and war-exposed civilian children in Nepal. Global mental health (Cambridge, England). 2018;5(101659641):e9.

Bile KM, Hafeez A, Kazi GN, Southall D. Protecting the right to health of internally displaced mothers and children: the imperative of inter-cluster coordination for translating best practices into effective participatory action. Eastern Mediterranean Health Journal. 2011;17(12):981-9.

Bosmans M, Gonzalez F, Brems E, Temmerman M. Dignity and the right of internally displaced adolescents in Colombia to sexual and reproductive health. Disasters. 2012;36(4):617-34.

Casey SE, Gallagher MC, Makanda BR, Meyers JL, Vinas MC, Austin J. Care-seeking behavior by survivors of sexual assault in the Democratic Republic of the Congo. American Journal of Public Health. 2011;101(6):1054-5.

Casey SE, Larsen MM, McGinn T, Sartie M, Dauda M, Lahai P. Changes in HIV/AIDS/STI knowledge, attitudes, and behaviours among the youth in Port Loko, Sierra Leone. Global Public Health. 2006;1(3):249-63.

Casey SE, McNab SE, Tanton C, Odong J, Testa AC, Lee-Jones L. Availability of long-acting and permanent family-planning methods leads to increase in use in conflict-affected northern Uganda: Evidence from cross-sectional baseline and endline cluster surveys. Global Public Health. 2013;8(3):284-97.

Casey SE, Tshipamba M. Contraceptive availability leads to increase in use in conflict-affected Democratic Republic of the Congo: evidence from cross-sectional cluster surveys, facility assessments and service statistics. Conflict and health. 2017;11:2.

Cherri Z, Cuesta JG, Rodriguez-Llanes JM, Guha-Sapir D. Early marriage and barriers to contraception among syrian refugee women in lebanon: A qualitative study. International Journal of Environmental Research and Public Health. 2017;14(8):836.

Chukwumalu K, Gallagher MC, Baunach S, Cannon A. Uptake of postabortion care services and acceptance of postabortion contraception in Puntland, Somalia. Reproductive Health Matters. 2017;25(51):48-57.

Cohen RA. Common Threads: A recovery programme for survivors of gender based violence. Intervention: International Journal of Mental Health, Psychosocial Work & Counselling in Areas of Armed Conflict. 2013;11(2):157-68.

Culbert H, Tu D, O'Brien DP, Ellman T, Mills C, Ford N, et al. HIV treatment in a conflict setting: outcomes and experiences from Bukavu, Democratic Republic of the Congo. PLoS Med. 2007;4:e129.

Curry DW, Rattan J, Huang S, Noznesky E. Delivering high-quality family planning services in crisis-affected settings II: results. Global health, science and practice. 2015;3(1):25-33.

Doumbouya B, Zaho M, Adouko S, Komena E, Mensah-Maika B, Toure S. The challenge of maintaining continuum of care and support to PLHIV in health facilities located in military conflict zones in Ivory Coast. Journal of the International AIDS Society. 2012;15:267.

Duroch F, McRae M, Grais RF. Description and consequences of sexual violence in Ituri province, Democratic Republic of Congo. BMC International Health and Human Rights. 2011;11(1):5.

Duroch F, Schulte-Hillen C. Care for victims of sexual violence, an organization pushed to its limits: The case of Médecins Sans Frontières. International Review of the Red Cross; 2014.

Ehui E, Couitchere LS, Kouakou GA, Doumbia A, Kassi AN, Mossou CM, et al. Antiretroviral chemoprophylaxis in children and adolescents victims of rape in Abidjan. Medecine et maladies infectieuses. 2015;45(8):324-7.

Ellman T, Culbert H, Torres-Feced V. Treatment of AIDS in conflict-affected settings: a failure of imagination. Lancet. 2005;365 North American Edition(9456):278-80.

Erickson M, Goldenberg SM, Akello M, Muldoon KA, Muzaaya G, Shannon K. Structural determinants of dual contraceptive use among female sex workers in conflict-affected Gulu, Northern Uganda. Canadian Journal of Infectious Diseases and Medical Microbiology. 2015;26:83B.

Feldman S, Freccero J, Seelinger KT. Safe Haven: Sheltering Displaced Persons from Sexual and Gender-Based Violence. Case Study: Colombia. 2013.

Foster AM, Arnott G, Hobstetter M. Community-based distribution of misoprostol for early abortion: evaluation of a program along the Thailand-Burma border. Contraception. 2017;96(4):242-7.

Freccero J, Seelinger KT. Safe Haven: Sheltering Displaced Persons from Sexual and Gender-Based Violence. Case Study: Thailand. 2013.

Frljak A, Cengic S, Hauser M, Schei B. Gynecological complaints and war traumas. A study from Zenica, Bosnia-Herzegovina during the war. Acta obstetricia et gynecologicaScandinavica. 1997;76(4):350-4.

Garang PG, Odoi RA, Kalyango JN. Adherence to antiretroviral therapy in conflict areas: a study among patients receiving treatment from Lacor Hospital, Uganda. AIDS patient care and STDs. 2009;23(9):743-7.

Gedeon J, Hsue SN, Walsh M, Sietstra C, MarSan H, Foster AM. Assessing the experiences of intra-uterine device users in a long-term conflict setting: a qualitative study on the Thailand-Burma border. Conflict and health. 2015;9(1):6.

Goodrich S, Ndege S, Kimaiyo S, Some H, Wachira J, Braitstein P, et al. Delivery of HIV care during the 2007 post-election crisis in Kenya: a case study analyzing the response of the Academic Model Providing Access to Healthcare (AMPATH) program. Confl Health. 2013;7(1):25.

Gupta J, Falb KL, Lehmann H, Kpebo D, Xuan Z, Hossain M, et al. Gender norms and economic empowerment intervention to reduce intimate partner violence against women in rural Côte d'Ivoire: a randomized controlled pilot study. BMC international health and human rights. 2013;13:46-.

Gurman TA, Trappler RM, Acosta A, McCray PA, Cooper CM, Goodsmith L. 'By seeing with our own eyes, it can remain in our mind': qualitative evaluation findings suggest the ability of participatory video to reduce gender-based violence in conflict-affected settings. Health education research. 2014;29(4):690-701.

Hampton T. Innovative program offers HIV therapy to internally displaced persons in Uganda. JAMA: Journal of the American Medical Association. 2008;300(5):493-.

Hemhongsa P, Tasaneeyapan T, Swaddiwudhipong W, Danyuttapolchai J, Pisuttakoon K, Rienthong S, et al. TB, HIV-associated TB and multidrug-resistant TB on Thailand's border with Myanmar, 2006-2007. Tropical medicine & international health : TM & IH. 2008;13(10):1288-96.

Horn R, Seelinger KT. Safe Haven: Sheltering Displaced Persons from Sexual and Gender-Based Violence. Case Study: Kenya. 2013.

Huber D, Saeedi N, Samadi AK. Achieving success with family planning in rural Afghanistan. Bulletin of the World Health Organization. 2010;88(3):227-31.

Hustache S, Moro M-R, Roptin J, Souza R, Gansou GM, Mbemba A, et al. Evaluation of psychological support for victims of sexual violence in a conflict setting: results from Brazzaville, Congo. Int J Ment Health Syst. 2009;3(1):7-.

International Rescue Committee (IRC). Women's Protection and Livelihoods: Assistance to Central Africa Refugees and Chadian Returnees in Southern Chad. 2016.

International Rescue Committee (IRC). Cost Efficiency Analysis: Distributing Family Planning Materials. 2016.

Iyakaremye I, Mukagatare C. Forced migration and sexual abuse: experience of Congolese adolescent girls in Kigeme refugee camp, Rwanda. Health Psychology Report. 2016;4(3):261-71.

Kabakian-Khasholian T, Mourtada R, Bashour H, Kak FE, Zurayk H. Perspectives of displaced Syrian women and service providers on fertility behaviour and available services in West Bekaa, Lebanon. Reproductive health matters. 2017;25(sup1):75-86.

Kaiser R, Kedamo T, Lane J, Kessia G, Downing R, Handzel T, et al. HIV, syphilis, herpes simplex virus 2, and behavioral surveillance among conflict-affected populations in Yei and Rumbek, southern Sudan. AIDS (London, England). 2006;20(6):942-4.

Kiboneka A, Nyatia RJ, Nabiryo C, Anema A, Cooper CL, Fernandes KA, et al. Combination antiretroviral therapy in population affected by conflict: outcomes from large cohort in northern Uganda. BMJ. 2009;338:201.

Kim AA, Malele F, Kaiser R, Mama N, Kinkela T, Mantshumba J, et al. HIV infection among internally displaced women and women residing in river populations along the Congo River, Democratic Republic of Congo. AIDS & Behavior. 2009;13(5):914-20.

Kinaro J, Ali TEM, Schlangen R, Mack J. Unsafe abortion and abortion care in Khartoum, Sudan. Reproductive health matters. 2009;17(34):71-7.

Kisindja RM, Kimona C, Etoy M, Dorme F, Benfield N. Family planning knowledge and use among women in camps for internally displaced people in the Democratic Republic of the Congo. International journal of gynaecology and obstetrics: the official organ of the International Federation of Gynaecology and Obstetrics. 2017;138(3):256-60.

Krause S, Williams H, Onyango MA, Sami S, Doedens W, Giga N, et al. Reproductive health services for Syrian refugees in Zaatri Camp and Irbid City, Hashemite Kingdom of Jordan: an evaluation of the Minimum Initial Services Package. Conflict and health. 2015;9(Suppl 1 Taking Stock of Reproductive Health in Humanitarian):S4.

Lee RB. Delivering maternal health care services in an internal conflict setting in Maguindanao, Philippines. Reproductive health matters. 2008;16(31):65-74.

Lilleston P, Winograd L, Ahmed S, Salame D, Al Alam D, Michelis I, et al. Reaching Refugee Survivors of Gender-Based Violence: Evaluation of a Mobile Approach to Service Delivery in Lebanon. 2016.

LokoRoka J, Van den Bergh R, Au S, De Plecker E, Zachariah R, Manzi M, et al. One size fits all? Standardised provision of care for survivors of sexual violence in conflict and post-conflict areas in the Democratic Republic of Congo. PLoS ONE. 2014;9(10):e111096.

Lokuge K, Shah T, Pintaldi G, Thurber K, Martinez-Viciana C, Cristobal M, et al. Mental health services for children exposed to armed conflict: Medecins Sans Frontieres' experience in the

Democratic Republic of Congo, Iraq and the occupied Palestinian territory. Paediatrics and international child health. 2013;33(4):259-72.
MalemoKalisya L, Lussy Justin P, Kimona C, Nyavandu K, Mukekulu Eugenie K, Jonathan KML, et al. Sexual violence toward children and youth in war-torn eastern democratic Republic of Congo. PLoS ONE. 2011;6(1):e15911.
Mankuta D, Aziz-Suleyman A, Yochai L, Allon M. Field evaluation and treatment of short-term psycho- medical trauma after sexual assault in the Democratic Republic of Congo. Israel Medical Association Journal. 2012;14(11):653-7.
Mayaud P. The challenge of sexually transmitted infections control for HIV prevention in refugee settings: Rwandan refugees in Tanzania. Transactions of the Royal Society of Tropical Medicine and Hygiene. 2001;95(2):121-4.
Mayaud P, Msuya W, Todd J, Kaatano G, West B, Begkoyian G, et al. STD rapid assessment in Rwandan refugee camps in Tanzania. Genitourinary medicine. 1997;73(1):33-8.
McGinn T, Allen K. Improving refugees' reproductive health through literacy in Guinea. Global Public Health. 2006;1(3):229-48.
McGinn T, Austin J, Anfinson K, Amsalu R, Casey SE, Fadulalmula SI, et al. Family planning in conflict: results of cross-sectional baseline surveys in three African countries. Conflict and health. 2011;5:11.
Mendelsohn JB, Schilperoord M, Spiegel P, Balasundaram S, Radhakrishnan A, Lee CK, et al. Is forced migration a barrier to treatment success? Similar HIV treatment outcomes among refugees and a surrounding host community in Kuala Lumpur, Malaysia. AIDS and behavior. 2014;18(2):323-34.
Mitike G, Deressa W. Prevalence and associated factors of female genital mutilation among Somali refugees in eastern Ethiopia: A cross-sectional study. BMC public health. 2009;9:264.
Mogga R. Addressing gender based violence and psychosocial support among South Sudanese refugee settlements in northern Uganda. Intervention. 2017;15(1):9-16.
Morren G, Van den Boogaard W, Dominguez E. Management of Obstetric Fistula in Burundi: The experience from a multidisciplinary approach over five years. 2016.
Morrison V. Contraceptive need among Cambodian refugees in Khao Phlu camp. International family planning perspectives. 2000;26(4):188-92.
Msuya W, Mayaud P, Mkanje R, Grosskurth H. Taking early action in emergencies to reduce the spread of STDs and HIV. Africa health. 1996;18(5):24.
Mukwege D, Berg M. A Holistic, Person-Centred Care Model for Victims of Sexual Violence in Democratic Republic of Congo: The Panzi Hospital One-Stop Centre Model of Care. PLoS medicine. 2016;13(10):e1002156.
Nattabi B, Li J, Thompson SC, Orach CG, Earnest J. Family planning among people living with HIV in post-conflict Northern Uganda: A mixed methods study. Conflict and health. 2011;5:18.
O'Brien DP, Mills C, Hamel C, Ford N, Pottie K. Universal access: the benefits and challenges in bringing integrated HIV care to isolated and conflict affected populations in the Republic of Congo. Confl Health. 2009;3:1-10.
O'Callaghan P, McMullen J, Shannon C, Rafferty H, Black A. A randomized controlled trial of trauma-focused cognitive behavioral therapy for sexually exploited, war-affected Congolese girls. Journal of the American Academy of Child and Adolescent Psychiatry. 2013;52(4):359-69.
O'Laughlin KN, Kasozi J, Walensky RP, Parker RA, Faustin ZM, Doraiswamy S, et al. Clinic-based routine voluntary HIV testing in a refugee settlement in Uganda. Journal of Acquired Immune Deficiency Syndromes. 2014;67(4):409-13.
Paik K. Strong Girls, Powerful Women: Program Planning and Design for Adolescent Girls in Humanitarian Settings. 2014.
Palmer JJ, Storeng KT. Building the nation's body: The contested role of abortion and family planning in post-war South Sudan. Social science & medicine. 2016;168:84-92.

Reid T, van Engelgem I, Telfer B, Manzi M. Providing HIV care in the aftermath of Kenya's post-election violence Medecins Sans Frontieres' lessons learned January - March 2008. Conflict and health. 2008;2:15-.

Rodger AJ, Toole M, Lalnuntluangi B, Muana V, Deutschmann P. DOTS-based tuberculosis treatment and control during civil conflict and an HIV epidemic, Churachandpur District, India. Bulletin of the World Health Organization. 2002;80(6):451-6.

Rosenberg JS, Bakomeza D. Let's talk about sex work in humanitarian settings: piloting a rights-based approach to working with refugee women selling sex in Kampala. Reproductive Health Matters. 2017;25(51):95-102.

Rowley EA, Spiegel PB, Tunze Z, Mbaruku G, Schilperoord M, Njogu P. Differences in HIV-related behaviors at Lugufu refugee camp and surrounding host villages, Tanzania. Conflict and health. 2008;2:13.

Rutta E, Gongo R, Mwansasu A, Mutasingwa D, Rwegasira V, Kishumbu S, et al. Prevention of mother-to-child transmission of HIV in a refugee camp setting in Tanzania. Global Public Health. 2008;3(3):62-76.

Salami O, Buzu A, Nzeme C. High level of adherence to HAART among refugees and internally displaced persons on HAART in western equatorial region of Southern Sudan. Journal of the International AIDS Society. 2010;13((Salami, Buzu, Nzeme) Tambura Hospital, International Medical Corps, Yambio, Sudan):no pagination.

Shaikh MA. Nurses' use of global information systems for provision of outreach reproductive health services to internally displaced persons. Prehospital & Disaster Medicine. 2008;23:s35-8.

Shamomesh M, Shamanesh M. The prevalence of urogenital Chlamydia trachomatis infection in a refugee community in El Salvador. International journal of STD & AIDS. 1994;5(5):381-2.

Somigliana E, Sabino A, Schrettenbrunner C, Nkurunziza R, Okello E, Manenti F. A comprehensive and integrated project to improve reproductive health at Oyam district, northern Uganda: Insights from maternal death review at the district hospital. Archives of gynecology and obstetrics. 2011;283(3):645-9.

Tanabe M, Robinson K, Lee CI, Leigh JA, Htoo EM, Integer N, et al. Piloting community-based medical care for survivors of sexual assault in conflict-affected Karen State of eastern Burma. Conflict and health. 2013;7(1):12.

Tanaka Y, Kunii O, Hatano T, Wakai S. Knowledge, attitude, and practice (KAP) of HIV prevention and HIV infection risks among Congolese refugees in Tanzania. Health Place. 2008;14(3):434-52.

Tanner S, O'Conner M. A Safe Place to Shine: Creating Opportunities and Raising Voices of Adolescent Girls in Humanitarian Settings 2017 October 2017.

Tayler-Smith K, Zachariah R, Hinderaker SG, Manzi M, De Plecker E, Van Wolvelaer P, et al. Sexual violence in post-conflict Liberia: survivors and their care. Tropical medicine & international health : TM & IH. 2012;17(11):1356-60.

Tousaw E, La RK, Arnott G, Chinthakanan O, Foster AM. "Without this program, women can lose their lives": migrant women's experiences with the Safe Abortion Referral Programme in Chiang Mai, Thailand. Reproductive Health Matters. 2017;25(51):58-68.

Tran NT, Harker K, Yameogo WME, Kouanda S, Millogo T, Menna ED, et al. Clinical outreach refresher trainings in crisis settings (S-CORT): clinical management of sexual violence survivors and manual vacuum aspiration in Burkina Faso, Nepal, and South Sudan. Reproductive Health Matters. 2017;25(51):103-13.

United Nations Children's Fund (UNICEF). Evaluation of UNICEF Programmes to Protect Children in Emergencies: Pakistan Country Case Study 2013.

United Nations Children's Fund (UNICEF). The UnicefResponse to the Crisis in the Central African Republic: Final Report 2016.

United Nations Children's Fund (UNICEF). Multi-Country Real Time Evaluation of UNICEF Gender-based Violence in Emergencies Programmes: Central African Republic Country Report. Child Protection Section PD; 2016.

United Nations Children's Fund (UNICEF). Multi-Country Real Time Evaluation of UNICEF Gender-based Violence in Emergencies Programme: Somalia Country Report. Child Protection Section PD; 2016.

United Nations Children's Fund (UNICEF). Evaluation of UNICEF Programmes to Protect Children in Emergencies: Democratic Republic of the Congo Case Study. Office E; 2013.

United Nations Children's Fund (UNICEF). Multi-Country Real Time Evaluation of UNICEF Gender-based Violence in Emergencies Programme: Lebanon Country Report. 2016.

United Nations Children's Fund (UNICEF). Multi-Country Real Time Evaluation of UNICEF Gender-based Violence in Emergencies Programmes: Pakistan Country Report. Child Protection Section PD; 2016.

United Nations Children's Fund (UNICEF). Multi-Country Real Time Evaluation of UNICEF Gender-based Violence in Emergencies Programmes: South Sudan Country Report. Child Protection Section PD; 2016.

United Nations High Commissioner for Refugees (UNHCR). Cash-based Interventions for Health programmes in Refugee Settings: A REVIEW. 2015.

United Nations High Commissioner for Refugees (UNHCR). Sustaining Results: A 9-month post-deployment impact assessment of the Senior Protection Officer (SGBV) in Mahama Camp, Rwanda. 2018.

United Nations Population Fund (UNFPA). Adolescent Girls in Disaster & Conflict: Interventions for Improving Access to Sexual and Reproductive Health Services 2016 August 2016.

United Nations Population Fund (UNFPA). Humanitarian Action 2017 Overview New York, NY; 2017.

United Nations Population Fund (UNFPA), UNICEF. 2017 Annual Report: UNFPA-UNICEF Global Programme to Accelerate Action to End Child Marriage 2018 August 2018.

Viswanathan K, Hansen PM, Rahman MH, Steinhardt L, Edward A, Arwal SH, et al. Can community health workers increase coverage of reproductive health services? Journal of Epidemiology and Community Health (1979-). 2012;66(10):894-900.

Von Roenne A, Von Roenne F, Kollie S, Swaray Y, Sondorp E, Borchert M. Reproductive health services for refugees by refugees: An example from Guinea. Disasters. 2010;34(1):16-29.

Vu A, Wirtz AL, Bundgaard S, Nair A, Luttah G, Ngugi S, et al. Feasibility and acceptability of a universal screening and referral protocol for gender-based violence with women seeking care in health clinics in Dadaab refugee camps in Kenya. Global mental health (Cambridge, England). 2017;4(101659641):e21.

Wayte K, Zwi AB, Belton S, Martins J, Martins N, Whelan A, et al. Conflict and Development: Challenges in Responding to Sexual and Reproductive Health Needs in Timor-Leste. Reproductive health matters. 2008;16(31):83-92.

West L, Isotta-Day H, Ba-Break M, Morgan R. Factors in use of family planning services by Syrian women in a refugee camp in Jordan. Journal of Family Planning and Reproductive Health Care. 2017;43(2):96-102.

Wirtz AL, Glass N, Pham K, Perrin N, Rubenstein LS, Singh S, et al. Comprehensive development and testing of the ASIST-GBV, a screening tool for responding to gender-based violence among women in humanitarian settings. Conflict and health. 2016;10:7.

Women's Refugee Commission (WRC). Optimizing Benefits and Mitigating Risks of Integrating Cash-Based Initiatives and GBV Programming: Case Studies from Irbid and Mafraq, Jordan 2018.

YiwezaTshipala D, Cornier N, Gounongbe M, Petros G, Assouan I, Bilguissa D, et al. Ensuring continuity of antiretroviral therapy among displaced populations during Ivorian post-election

violence, 2011. Journal of the International AIDS Society. 2012;15((Bilguissa) UNHCR Consultant, Public Health and HIV Section, Paris, France):266-7.
Yoshikawa L. Integrating Cash Transfers into Gender-based Violence Programs in Jordan: Benefits, Risks and Challenges. 2015 November 2015.
